# SHARC meets TEQUILA: mixed quantum-classical dynamics on a quantum computer using a hybrid quantum-classical algorithm†

**DOI:** 10.1039/d4sc04987j

**Published:** 2024-11-28

**Authors:** Eduarda Sangiogo Gil, Markus Oppel, Jakob S. Kottmann, Leticia González

**Affiliations:** a Faculty of Chemistry, Institute of Theoretical Chemistry, Universität Wien A-1090 Vienna Austria eduarda.sangiogo.gil@univie.ac.at leticia.gonzalez@univie.ac.at; b Institute for Computer Science, Center for Advanced Analytics and Predictive Sciences, Universität Augsburg Augsburg Germany

## Abstract

Recent developments in quantum computing are highly promising, particularly in the realm of quantum chemistry. Due to the noisy nature of currently available quantum hardware, hybrid quantum-classical algorithms have emerged as a reliable option for near-term simulations. Mixed quantum-classical dynamics methods effectively capture nonadiabatic effects by integrating classical nuclear dynamics with quantum chemical computations of the electronic properties. However, these methods face challenges due to the high computational cost of the quantum chemistry part. To mitigate the computational demand, we propose a method where the required electronic properties are computed through a hybrid quantum-classical approach that combines classical and quantum hardware. This framework employs the variational quantum eigensolver and variational quantum deflation algorithms to obtain ground and excited state energies, gradients, nonadiabatic coupling vectors, and transition dipole moments. These quantities are used to propagate the nonadiabatic molecular dynamics using the Tully's fewest switches surface hopping method, although the implementation is also compatible with other molecular dynamics approaches. The approach, implemented by integrating the molecular dynamics program package SHARC with the TEQUILA quantum computing framework, is validated by studying the *cis*–*trans* photoisomerization of methanimine and the electronic relaxation of ethylene. The results show qualitatively accurate molecular dynamics that align with experimental findings and other computational studies. This work is expected to mark a significant step towards achieving a “quantum advantage” for realistic chemical simulations.

## Abstract 2

We present a hybrid quantum-classical approach that combines quantum and classical hardware to simulate the excited state dynamics of molecules.

## Graphical abstract

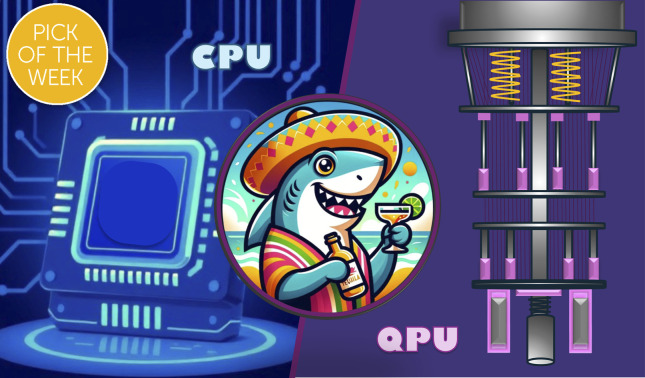

## 1 Introduction

As suggested by Feynman^1^ in the 1980s, simulating quantum mechanical systems on a quantum computer should be more efficient than running the same computation on a classical computer. Especially quantum chemical simulations have emerged as particular promising applications.^2–5^ Current quantum computing hardware classify as “noisy intermediate scale quantum technology” (NISQ) devices.^6,7^ The limited amount of available qubits and the still open problem of efficient error correction leaves the complete simulation of large and complex physical and chemical systems a distant goal. Hybrid quantum-classical algorithms, where only parts of the problem are solved on quantum devices^8^ are expected to be crucial in near-term quantum simulations,^6^

Over the past decade the variational quantum eigensolver (VQE) has become the de-facto standard hybrid quantum-classical algorithm for quantum chemical calculations.^9–11^ Despite its initial design for computing electronic ground states, different variations for calculating electronically excited states have been developed. Especially penalty-based optimization^12–15^ and quantum equations of motion (qEOM)^16^ approaches have shown promising results.

Mixed quantum-classical dynamics methods, which simulate nonadiabatic processes by combining classical trajectory propagation for the nuclear dynamics with a quantum mechanical treatment of the electrons, are effective in capturing nonadiabatic effects through a feedback algorithm that integrates the electronic and nuclear subsystems. Using a classical treatment of the nuclei allows for a favorable scaling with the system size. The significant bottleneck in these simulations is the computation of the electronic properties. The most common approach in mixed quantum-classical dynamics is to compute the electronic properties on-the-fly, *i.e.*, at each timestep of the dynamics. Advantageously, this does not require pre-computed multidimensional potential energy surfaces. Still the computational cost of these dynamics simulations is heavily dependent on the accuracy of the electronic property calculations, making it necessary to find a compromise between accuracy and feasibility.

Moving portions of the quantum chemical computations for the electronic structure onto quantum computers should accelerate the overall computational effort. In this work, we utilize hybrid quantum-classical algorithms to compute electronic properties on-the-fly during mixed quantum-classical dynamics. Recently, some studies^17,18^ have demonstrated the ability of hybrid quantum-classical algorithms to accurately predict conical intersections, which is relevant to perform nonadiabatic dynamics.

While previous studies have explored the application of hybrid quantum-classical algorithms in the context of excited state dynamics, they are often limited to toy models and very small molecular systems.^19–24^ In contrast, we focus on a hybrid quantum-classical framework able to perform nonadiabatic dynamics for polyatomic molecules with complex and interesting photochemistry.

Although our approach is implemented using Tully's fewest switches surface-hopping (SH) method,^25^ it can be easily extended to other molecular dynamical approaches.

The ground state electronic properties are computed using a standard VQE algorithm, while excited state properties are obtained through penalty-based optimization, which ensure the orthogonality of the wavefunction with previously calculated electronic states, thus allowing to subsequently compute multiple electronically excited states. Our approach is implemented by interfacing our molecular dynamics program package SHARC^26,27^ with the TEQUILA quantum computing framework.^28^

Recently, Tavernelli and co-workers employed the quantum subspace expansion and quantum equation-of-motion algorithms to simulate the SH dynamics of a hydrogen atom colliding with a hydrogen molecule, a system comprising three nuclei and three electrons.^22^ In the subspace expansion framework, excited states are represented as linear combinations of intermediary states, typically derived from the approximate ground-state wave function. While effective, the use of truncated subspaces is not strictly variational for excited states, which can sometimes limit accuracy. In contrast, we have employed a penalty-based method (see Section 2.2). This is not only simpler to implement, but also eliminates the dependence on the number of excited states required for accuracy. In our approach, each additional state is calculated variationally through a penalty function, enforcing orthogonality to previously computed states and maintaining precision without the limitations of truncated subspaces.

As we are bridging both the nonadiabatic dynamics and quantum information communities, the next section explains how electronic properties are computed using the VQE algorithm. In the following, we provide a detailed outline of the dynamics involved and illustrate how these electronic properties, obtained through the VQE, enter the nuclear dynamical procedure. Finally, we showcase our approach by investigating the nonadiabatic dynamics of two systems: the *cis*–*trans* photoisomerization of methanimine and the ultrafast electronic relaxation of ethylene.

## 2 Methods

### 2.1 Ground state energies and gradients

We utilized the hybrid quantum-classical algorithm, the VQE, to compute the ground state energies and gradients during the dynamics. The VQE was first introduced by Peruzzo *et al.*^9^ and subsequently extended later in ref. 10. It is based on the variational principle, which involves optimizing an upper bound for the lowest possible expectation value of an observable with respect to the trial wavefunction.^11^ Specifically, given a Hamiltonian 
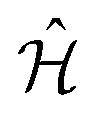
 and a trial wavefunction *Ψ*(*θ*), the ground state energy associated with this Hamiltonian, *E*_gs_, is bounded by:1
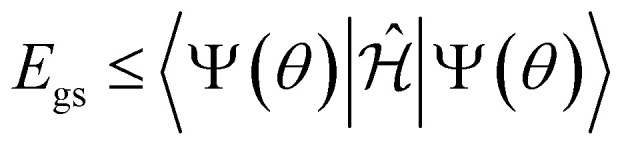
where the trial wavefunction *Ψ*(*θ*) is assumed to be normalized, and *θ* is the set of variational parameters. Therefore, the objective of the VQE is to find the set of *θ* that minimizes the expectation value of 
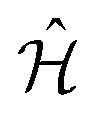
. The trial wavefunction is constructed using a quantum circuit, known as the ansatz. This involves applying a series of quantum gates (unitary operations) to a reference state *Ψ*_0_. Thus, the trial wavefunction *Ψ*(*θ*) can be represented as **U**(*θ*)|*Ψ*_0_〉, where **U**(*θ*) denotes the ansatz.

The VQE algorithm has shown to be an interesting approach for solving quantum chemistry problems. In this context, the molecular Hamiltonian in second quantization can be written as:2
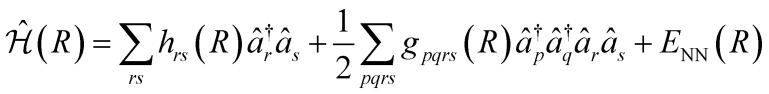
where *h*_*rs*_ and *g*_*pqrs*_ denote the one- and two-electron integrals, respectively, and are commonly obtained from a Hartree–Fock (HF) calculation, which is usually performed on a classical device. The collective vector of nuclear coordinates *R* = (*R*_1_, *R*_2_, …, *R*_*N*_) of *N* nuclei in *R*^3*N*^ simply parametrizes the electronic Hamiltonian. The position vector of a single nucleus *I* ∈ {1, …, *N*} is denoted by *R*_*I*_ = (*R*_*Ix*_, *R*_*Iy*_, *R*_*Iz*_). The operators 
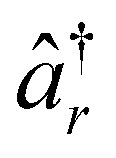
 and *â*_*r*_ represent the fermionic creation and annihilation operators for electrons in HF spin–orbitals. The indices *r*, *s*, *t*, *u* are used to label general (occupied or virtual) molecular orbitals. The term *E*_NN_(*R*) describes the nuclear repulsion energy.

Observables suitable for direct measurements on a quantum device are tensor products of spin operators (Pauli operators). To achieve this, the Hamiltonian of eqn (2) can be transformed into a qubit operator by applying, for example, the Jordan–Wigner transformation,^29^ so that the molecular Hamiltonian 
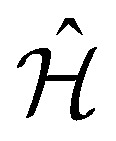
 can be expressed as:3
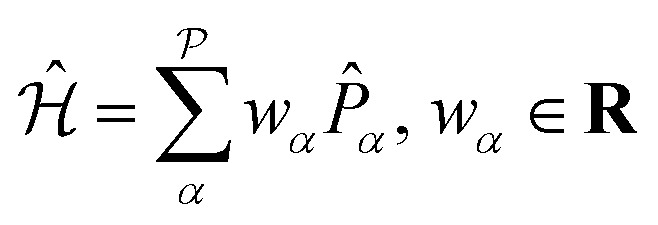
where *w*_*α*_ is the set of weights and 
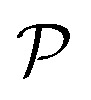
 the number of Pauli strings *P̂*_*α*_ in the Hamiltonian.

Therefore, the expectation value of the molecular Hamiltonian with the VQE can be calculated with:4
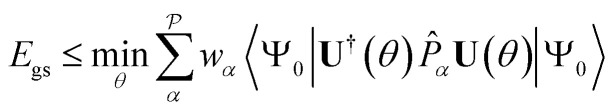
where the hybrid nature of the VQE algorithm becomes clearly apparent: each term *E*_*P*_*α*__ = 〈*Ψ*_0_|**U**^†^(*θ*)*P̂*_*α*_**U**(*θ*)|*Ψ*_0_〉 corresponds to the expectation value of a Pauli string *P̂*_*α*_ and can be computed on a quantum device, while the summation and minimization 
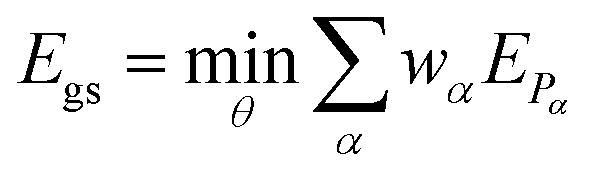
 is computed on a conventional computer. The reference wavefunction |*Ψ*_0_〉 is often the HF wavefunction. The evaluation of the expectation value in eqn (3) often becomes the computational bottleneck of the VQE procedure, as the number of Pauli strings in a naive decomposition scales quartic with respect to the number of orbitals. There exist however methods to group the Pauli strings into commuting cliques^30–32^ that mitigates this issue – the number of diagonal bases in the ethylene molecule used below is for example reduced from 34 (native Pauli bases) to just 3 (commuting Pauli cliques) using the method of ref. 31. For a full treatment (6-31G basis), the reduction would be from 73 089 to 552.

The quality of the energy obtained from eqn (4) also depends on the ansatz. Several ansätze have been proposed in recent years for VQE applications in quantum chemistry. Among the most commonly used is the Unitary Coupled Cluster (UCC) approach and its extensions.^33–36^ Other popular choices include the Hardware Efficient Ansatz (HEA),^37^ and the anti-Hermitian contracted Schrödinger equation (ACSE) ansatz, which has been effectively used in contracted quantum eigensolvers (CQE) on quantum computers.^38–40^ In this work, we have adopted a special type of UCC, termed k-UpCCGSD.^41^ This ansatz presents a good compromise between accuracy and cost, showing better scaling than UCCSD and UGCCSD.

The derivatives of the energy with respect to the nuclear coordinates for a given nucleus *I*, 
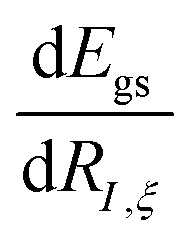
 (energy gradients), where *ξ* ∈ {*x*, *y*, *z*}, are needed to calculate the forces acting on the nuclei. The total derivative is given explicitly by:5
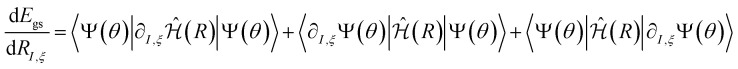
with 
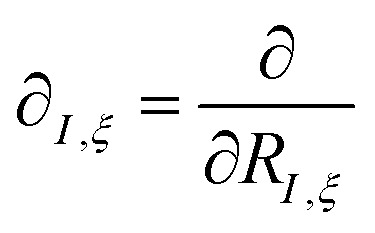
. The first term in eqn (5) is the Hellmann–Feynman force, while the second and third terms are the Pulay forces. In our implementation, we approximated the energy gradients by considering only the Hellmann–Feynman term:6
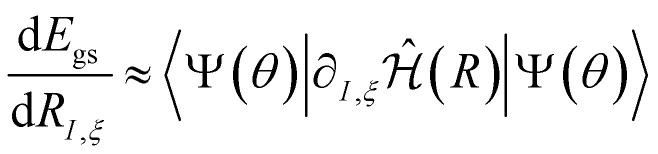
where the gradients of the molecular Hamiltonian were calculated using the central difference formula with a shift parameter of 0.001 angstroms.

We note that this approximation has also been used in other studies that disregard Pulay forces.^19–22^ Already in 2010, Foley and Mazziotti^42^ demonstrated efficient geometry optimization and frequency analysis by using only the Hellmann–Feynman component for the ACSE. Pulay forces are considered essential for correcting errors arising from basis set incompleteness.^43^ Consequently, a complete basis set would result in net zero Pulay forces. Since we did not use a complete basis set, we assessed the accuracy of neglecting Pulay forces by comparing gradients calculated solely using the Hellmann–Feynman term with those obtained using the central difference formula of the energy (which includes both Pulay and Hellmann–Feynman terms) across various geometries. Generally, the gradients from both methods were nearly identical, indicating that neglecting Pulay forces is valid in this context. Further details and discussions on these results are provided in Section S1 of the ESI.†

It is also worth mentioning that another alternative, which allows forces to be computed solely using the Hellmann–Feynman term, is to use a perturbation-dependent basis set.^44^

### 2.2 Excited state energies and gradients

The excited states can be calculated similarly to the method used by the VQE, but instead of minimizing the energy, a cost function is minimized.^12–15^ This method is known as variational quantum deflation (VQD). The definition of the cost function depends on the target state and the target system. For instance, the energy of the first excited state can be calculated using the cost function:7
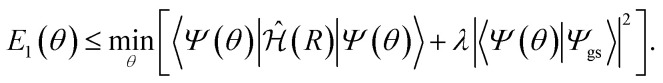
Here, *Ψ*_gs_ is the ground state wavefunction that was previously computed with VQE, and *λ* is a hyperparameter that must be specified before the VQD calculation. The second term in eqn (7) enforces the constraint of searching the subspace orthogonal to the ground state. Following the ref. 45, we can express the overlap punishment as another expectation value

8 |〈*Ψ*(*θ*)|*Ψ*_gs_〉|^2^ = 〈*Φ*(*θ*)|*P*_0_|*Φ*(*θ*)〉

with |*Φ*(*θ*)〉 = **U**(*θ*)^†^|*Ψ*(*θ*)〉 and *P*_0_ = |0〉〈0| with the all-qubit zero state |0〉. The parameter *λ* should be sufficiently large, ideally larger than the energy difference between the ground and the excited state. However, if *λ* is too large, it could lead to the selection of an undesired higher excited state.^46^ A good initial guess for *λ* could be the magnitude of the ground state energy obtained with VQE – which is sufficient if the excited state is bound (*i.e.* has negative energy)^45^ – and this is the *λ* used in this work. In addition, the ansatz adopted in this work, unlike the HEA, prevents spin contamination since the number of electrons and the spin state are conserved. Therefore, no additional considerations, such as including penalty terms in the cost function (eqn (7)) are needed.

We need to stress that the VQD faces significant challenges due to its sequential computation requirement. Each eigenstate computation depends on the accurate deflation of previously found states, leading to increased computational overhead and potential optimization difficulties. This sequential nature can complicate the optimization process, making it harder to accurately identify and optimize higher excited states. Consequently, VQD may miss some states or converge to suboptimal solutions, particularly in more complex systems or under noisy quantum conditions.

The gradients of the excited state energies are obtained similarly to those for the ground state (see eqn (6)). Once we have the gradient of the molecular Hamiltonian, 
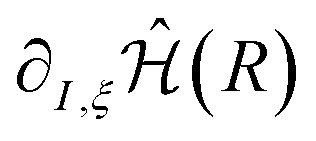
, the gradients of all the states can be easily calculated as the expectation value of this derivative for the wavefunctions obtained from the VQE or VQD algorithms. Therefore, the computational cost for the computation of the gradients does not increase significantly with the number of states.

### 2.3 Surface hopping dynamics

The time evolution of a (non-relativistic) molecular system is determined by the time-dependent Schrödinger equation (TDSE), 
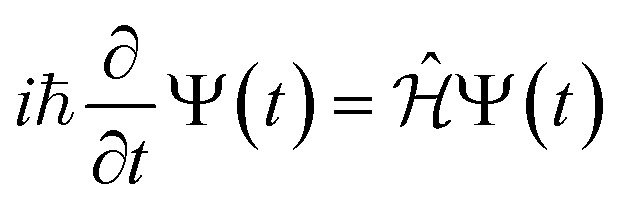
. Unfortunately, solving the TDSE and simulating quantum dynamics in molecules remains a fundamentally difficult problem due to the non-local nature of quantum mechanics and the associated exponential scaling of the computational effort with the number of degrees of freedom. One popular and effective approximation is to describe the nuclear motion using classical mechanics instead of quantum mechanics. This approach is motivated by the favorable scaling of classical mechanics with system size. The classical approximation yields reasonable results in cases where quantum mechanical effects, such as tunneling or interference, are negligible, and the energetic spacing between quantum levels is small compared to the kinetic energy. Since nuclei are much heavier than electrons, the spacing between quantum levels is usually much smaller for nuclear degrees of freedom than for electronic ones. Therefore, it is plausible to treat the nuclei with classical mechanics while treating the electrons with quantum mechanics. Based on this assumption, numerous quantum-classical methods have been developed over the past decades to address nonadiabatic processes. Among these methods, the SH scheme has emerged as one of the most widely used approaches.^47–50^ In this work, we used the Tully's fewest switches SH,^25^ but the approach presented here can be easily extended to others mixed quantum-classical dynamics approaches. The basic assumption is that during nonadiabatic dynamics, the nuclei move adiabatically for most of the time and only undergo nonadiabatic transitions for relatively short periods and in relatively small regions of the configuration space. Hence, it is pragmatically proposed that one could approximate the nonadiabatic transitions by instantaneous “hops” between adiabatic potential energy surfaces (PESs). In the SH approach, many trajectories are simulated independently, generating a statistical ensemble. The fraction of trajectories for each PES is used to mimic the population of each quantum state in realistic dynamical processes. In SH dynamics, the time evolution of the nuclei, *i.e.*, the classical degrees of freedom *R*, is performed by integrating Newton's equation with the potential being in a given adiabatic surface *E*_*m*_(*R*). Here, Newton's equation is solved using the velocity-Verlet algorithm.^51^ In this algorithm, the nuclear positions *R*_*I*,*ξ*_ and velocities *Ṙ*_*I*,*ξ*_ are updated from a time step, *t*, to the next step, *t* + Δ*t*, with the following equations:9
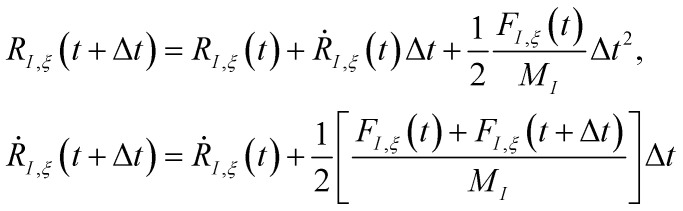
where *M*_*I*_ is the atomic mass of a given nucleus *I*, and the forces acting on the nuclei, *F*_*I*,*ξ*_, are the negative derivative of the electronic energy with respect to the nuclear coordinates, 
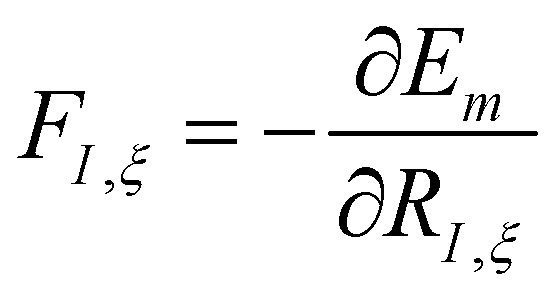
.

Besides the nuclei, the electronic wavefunction must be propagated as well. The electronic wavefunction is expressed as a sum over basis states:
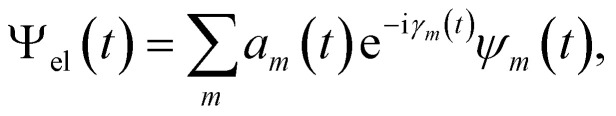
where *m* indexes the basis states |*ψ*_*m*_〉 (eigenstates of the electronic Hamiltonian), *a*_*m*_(*t*) are time-dependent coefficients, and *γ*_*m*_(*t*) represents the phase: 
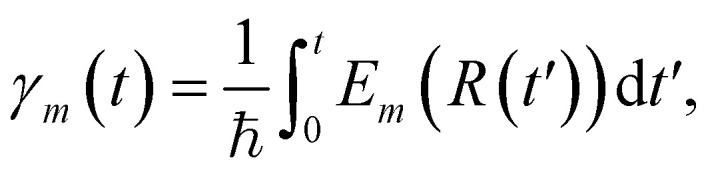
 with *E*_*m*_(*R*) being the adiabatic energy evaluated at the nuclear configuration *R*.

Incorporating *Ψ*_el_(*t*) into the TDSE yields the equation for the time evolution of the coefficients *a*_*m*_(*t*):10
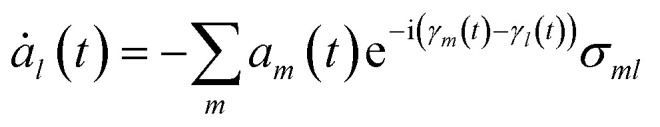
where *σ*_*ml*_ represents the time-derivative nonadiabatic coupling:11
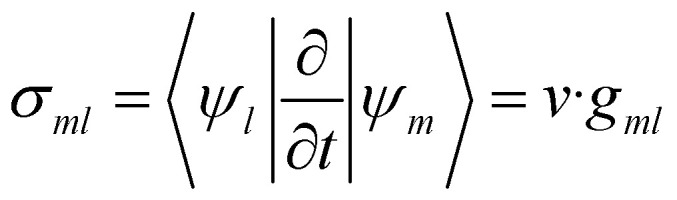
with *v* being the nuclear velocity, and *g*_*ml*_ the first-order nonadiabatic coupling (NAC) vector, defined as:12
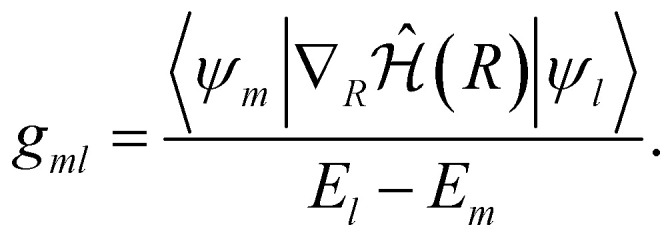
Within the VQE/VQD framework, the NAC vectors can be approximated as13
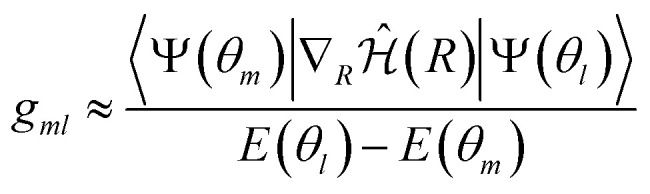
where *Ψ*(*θ*_*m*_) and *Ψ*(*θ*_*l*_) represent the wavefunctions optimized by VQE or VQD for states *m* and *l*, respectively, with corresponding energies *E*(*θ*_*m*_) and *E*(*θ*_*l*_). Once the energies and gradients are obtained, evaluating the NAC vectors is straightforward and present a similar computational cost to that of computing the gradients (see eqn (6)). A similar methodology for computing first-order NAC vectors was proposed in ref. 24.

However, NAC vectors can change rapidly, often necessitating very small time steps to avoid numerical issues, especially near narrow peaks found in NACs near strong interaction regions such as avoided crossings and conical intersections. Additionally, crossings between uncoupled states (“trivial crossings”) are invariably missed regardless of time step size.^52^ To circumvent these challenges, we employed the local diabatization scheme for integrating electronic coefficients.^53,54^ In this scheme, the NAC vectors are never explicitly computed. Instead, the wavefunction overlap matrix at two different time steps is required. The basic idea of the local diabatization is to resort to a “locally diabatic” representation, *i.e.*, to a set of electronic states that are specifically diabatic along the nuclear trajectory under consideration. By definition, the diabatic basis also spans the internal subspace and is connected with the adiabatic one by a unitary transformation:

14 |*η*〉 = |*ψ*〉*T*

where |*η*〉 and |*ψ*〉 are the wavefunctions in diabatic and adiabatic basis, respectively.

If *H* is the Hamiltonian in the diabatic basis (*H*_*kl*_ = 〈*η*_*k*_|*Ĥ*_el_|*η*_*l*_〉) and *E* the diagonal matrix of the electronic energies, we have

15 *HT* = *TE*.

The diabatic expansion of the time-dependent wavefunction is16
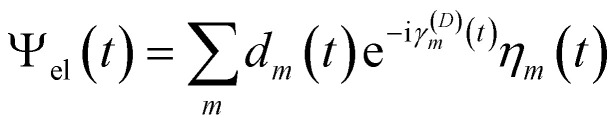
with 
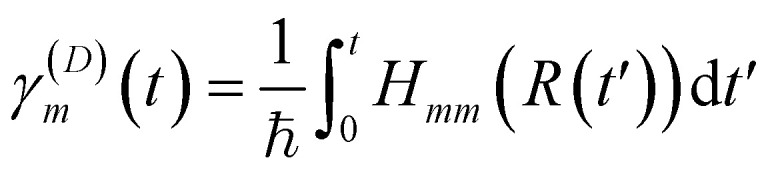
 and

17 *d* = *Ta*

where *d* and *a* are the matrices of the diabatic and adiabatic coefficients, respectively. Here, *H*_*mm*_ are the diagonal elements of the *Ĥ*_el_ matrix in the diabatic basis, *i.e.*, *H*_*mm*_ = 〈*η*_*m*_|*Ĥ*_el_|*η*_*m*_〉.

The diabatic basis in the local diabatization scheme is redefined at each time step. At the beginning of the time step, the transformation matrix is chosen to be the identity matrix, thus *η* ≡ *ψ*. By choosing the diabatic states to be constant within the time step considered, the dynamic couplings 〈*η*_*k*_|∂/∂*t*|*η*_*l*_〉 are eliminated. However, the coupling vanishes only along the advancement coordinate identified by the velocity vector *Ṙ*: in this sense the *η* are “locally” diabatic” for the given trajectory. Then, by inserting the electronic wavefunction expansion (16) into the TDSE, the time evolution of the diabatic coefficients is obtained:18
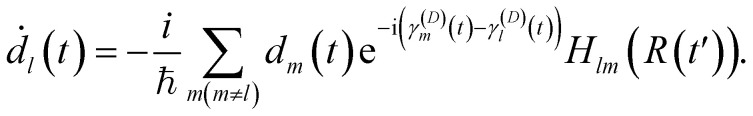
Within the local diabatization algorithm, the transformation matrix *T* is obtained by Löwdin orthonormalization of the wavefunction overlap matrix

19 *S*_*ml*_ (*t* + Δ*t*) = 〈*ψ*_*m*_(*t*)|*ψ*_*l*_(*t* + Δ*t*)〉.

Hence, from *T*, one obtains *H*(*t* + Δ*t*). The matrix *H* at intermediate times in the interval [*t*, *t* + Δ*t*] is attained by interpolation from *H*(*t*) to *H*(*t* + Δ*t*) (note that *H*(*t*) = *E*(*t*)). The essential point is that, unlike the NACs, the diabatic quantities (such as *H*) depend smoothly on the nuclear coordinates and can be easily and effectively interpolated. Then, the diabatic coefficients are obtained from eqn (18). Finally, the adiabatic expansion coefficients *a*(*t* + Δ*t*) are computed through the inverse of eqn (17).

In the present context, *ψ*_*m*_ represents the wavefunctions optimized by VQE or VQD for states *m* and *l*, denoted as *Ψ*(*θ*_*m*_) and *Ψ*(*θ*_*l*_), respectively. However, these wavefunctions correspond to different time steps, meaning that the molecular orbitals in the wavefunctions vary over time. However, to compute the overlaps in eqn (19), we must first convert the wavefunction into a “configuration interaction type” wavefunction. This process requires providing a list of determinants, their corresponding coefficients, and the molecular orbitals for both the bra and ket states. These overlaps are then computed using a classical algorithm.

The local diabatization scheme allows using larger time steps and naturally mitigates the trivial crossing issue.

#### 2.3.1 Initial conditions preparation

We showed how in SH the nuclear and electronic properties (adiabatic energies, energy's gradients and NACs) can be propagated from a time step *t* to the next step, *t* + Δ*t*. However, to start an SH trajectory, we need to provide: (i) the initial nuclear positions *R*; (ii) initial nuclear velocities *v*; (iii) the initial electronic state *m*; and (iv) the initial matrix of the electronic wavefunction coefficients *a*. This set of values is called the initial conditions.

The electronic wavefunction coefficients *a* and the initial state *m* depend on the excitation process. There are two techniques that are often used to sample *R* and *v*. The first one is called Wigner sampling and it computes an approximate phase space probability distribution function representing the ground vibrational state and then stochastically draws samples from this distribution. The advantage of Wigner sampling is that quantum effects like zero-point energy are adequately considered. However, it is often challenging to find an appropriate nuclear wavefunction for polyatomic molecules; thus, one usually resorts to the harmonic approximation, which works well for small and stiff molecules.^55,56^ An alternative to Wigner sampling is to run a long dynamics simulation in the ground state and randomly pick *R* and *v* from this trajectory. This approach does not represent quantum effects like zero-point energy well but works effectively for large, polyatomic systems with anharmonic or nonlinear modes and multiple local minima in the ground state PES.^57–59^ While the latter approach is easier to implement, as it only requires energies and gradients-which we have already demonstrated how to compute-we decided to use Wigner sampling, as we believe this method is more appropriate for the types of systems studied in this work. For Wigner sampling, one needs to provide the normal modes and their corresponding frequencies. This requires calculating the second derivatives of the energy with respect to the nuclear coordinates. Since obtaining the analytical solution for this derivative is not straightforward, and since we only need to compute these derivatives at a single point-specifically, the minimum of the ground state, we opted for a fully numerical approach using central finite differences to calculate second derivatives.

As we have sampled the quantities *R* and *v*, it is possible to find the electronic wavefunction coefficients *a* and the initial state *m*. In the simplest case, it is possible to set *a*_*m*_(0) = *δ*_*ml*_, with *l* being one of the excited states. This *l* can be simply defined by the user (*e.g.*, all trajectories start in *l* = 3, *i.e.*, the S_2_ state).

A more appropriate and popular approach is to perform a single point calculation for each sampled *R* and compute a selection probability based on the obtained excitation energies and oscillator strengths of each state20
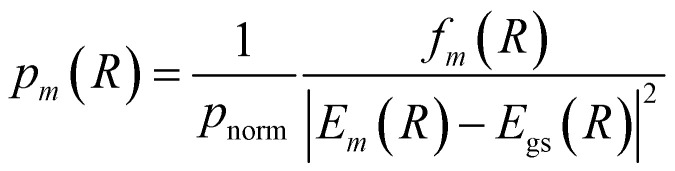
where *f*_*m*_ is the oscillator strength of state *m*, and *p*_norm_ is chosen arbitrarily. With these probabilities, one can stochastically select initial states. Additionally, some restrictions on |*E*_*m*_(*R*) − *E*_gs_(*R*)| are imposed to consider only a small excitation energy window. Therefore, our implementation also includes the calculation of transition dipole moments within the VQE/VQD framework. These calculations are necessary to determine oscillator strengths and to select the initial conditions accordingly.

### 2.4 Dynamics workflow

**Fig. 1:**
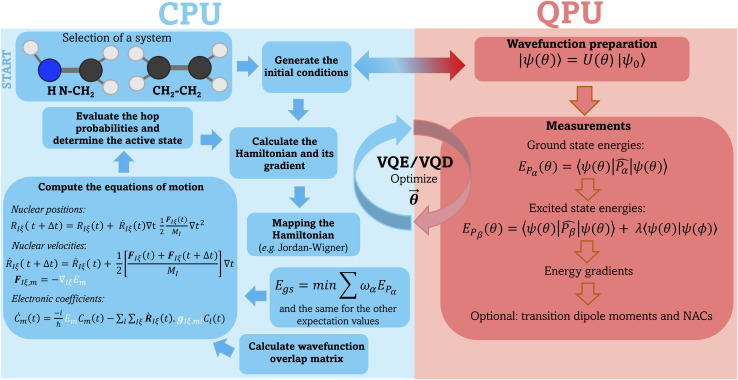
General workflow used to perform surface hopping dynamics in combination with the VQE/VQD algorithm. All parts of the simulations running on classical hardware (CPU) are depicted on the left hand side of the picture and shaded in blue. The preparation of the electronic wavefunction and the evaluation of the ground and excited state energies as well as the gradients and (optionally) transition dipole moments and NACs are carried out on the quantum device (QPU), shown on the right hand side of the picture, shaded in red. These data are then used by the classical hardware to propagate the nuclear dynamics (elements highlighted in white on the left panel).

In our implementation, the computation of the electronic properties such as energies, gradients, and transition dipole moments are carried out using the TEQUILA quantum computing software^28^ using the Qulacs^60^ quantum circuit simulation library. The overlap matrix between consecutive time steps (eqn (19)) is computed using the WFoverlap program,^61^ and the SH dynamics are executed using the SHARC package.^26,27^ After selecting the initial conditions, the workflow for each SH trajectory in the VQE/VQD framework is as follows:

(1) Calculate energies according to eqn (4) and (7).

(2) Compute gradients using eqn (6).

(3) Determine the overlap matrix between consecutive time steps (eqn (19)).

(4) Provide energies, gradients, and the wavefunction overlap matrix to the driver responsible for nuclear dynamics—the SHARC software in this case. This driver integrates the electronic TDSE, evaluates hopping probabilities, and computes new nuclear coordinates and velocities.

(5) Repeat steps 1–4 until reaching the desired trajectory endpoint. Fig. 1 illustrates a schematic representation of our workflow, highlighting the components performed on classical and quantum devices.

## 3 Applications of surface-hopping dynamics on quantum computers

**Fig. 2:**
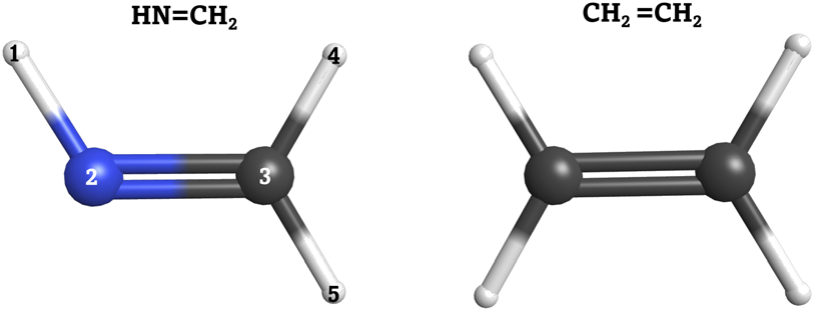
Left: Methanimine (HN=CH_2_). Right: Ethylene (CH_2_=CH_2_). Carbon (C) atoms in gray, nitrogen (N) atom in blue and hydrogen (H) atoms in white.

In order to validate our SH implementation on quantum computers, we applied it to two case studies: the *cis*–*trans* photoisomerization of methanimine (HN=CH_2_) and the electronic relaxation of ethylene (CH_2_=CH_2_). Both molecular systems are shown in Fig. 2. The small molecular size, ultrafast dynamics through a conical intersection, and massive conformational flexibility make these molecules suitable for ensure that our approach works as expected. Moreover, a large number of nonadiabatic dynamical studies have been carried out on these systems, employing various dynamical methods combined with different levels of theory to describe the PESs,^62–80^ which serve as reference. Both systems are also ideal to test our algorithm, as a proper description of the electronic structure problem only requires a small number of active orbitals. This is particularly important as the number of qubits required for the ansatz used is proportional to the number of active spin–orbitals.

### 3.1 Test case HN=CH_2_

#### 3.1.1 Computational details

The VQE/VQD electronic energies and wavefunctions of HN=CH_2_ were obtained using an active space of 4 electrons and 3 orbitals (6 spin–orbitals), requiring 6 qubits to construct the ansatz. The active space consisted of the n, π, and π* orbitals, warrantying that both nπ* and ππ* excitations were considered. A HF/6-31G wavefunction was used as a reference, calculated with PySCF.^81^ The Jordan–Wigner mapping^29^ was applied to generate the qubit Hamiltonian, and both VQE and VQD used the BFGS optimizer to minimize the parameters *θ*. In the first time step, the parameters *θ* were set to zero. However, for subsequent time steps, the initial *θ* values were those obtained from the optimization of the previous time step. This approach serves two purposes: first, it is expected that fewer interactions will be needed to optimize *θ* for the new geometry rendering the computations faster; second, and more importantly, it ensures that the VQD algorithm tracks the same set of states throughout the dynamics. For the first excited state, 6 angles were optimized, requiring an average over a single trajectory of 8.12 iterations (optimization steps) per nuclear time step (with a time step of 0.5 fs). For the second excited state, 13 angles were utilized, but only 7 were optimized, necessitating an average of 13.87 iterations. For the third excited state, 19 angles were involved, with 6 optimized, averaging 20.04 iterations per nuclear time step. Each nuclear time step involved a new calculation, resulting in varying numbers of interactions during the dynamics. As mentioned previously, the optimized angles from the previous time step were used as initial guesses for the next step, which reduced the number of interactions needed. Additionally, as expected, reducing the nuclear time step led to fewer iterations; for example, when the time step was reduced to 0.2 fs, the number of iterations was approximately halved.

**Fig. 3:**
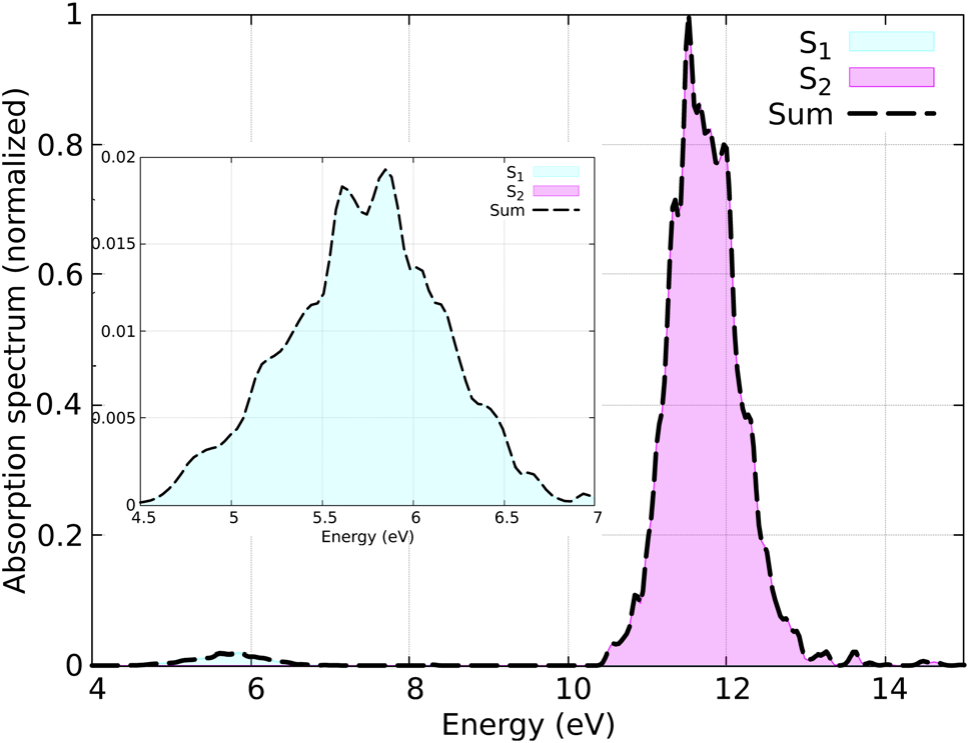
Simulated absorption spectrum of HN=CH_2_ calculated using 5000 geometries. The inset shows the nπ* band. Contributions from each adiabatic state are indicated, with the dashed line representing the total spectrum.

The distribution of initial coordinates and velocities was generated from a Wigner distribution, from which the spectrum shown in Fig. 3 is obtained. The starting conditions (geometries, velocities, and initial state) were selected according to the excitation window ranging from 5.5 eV to 6.0 eV, which corresponds to the excitation to the S_1_ state (nπ* excitation). The lowest three lying electronic states (S_0_–S_2_) were considered during the SH simulations. A total of 150 initial conditions were extracted from a nuclear ensemble of 5000 geometries. In total, 141 were used to analyze the dynamics; 9 trajectories were discarded because the total energy was not conserved by the end of the dynamics due to changing the orbitals in the active space, resulting in inconsistent PESs or numerical instabilities in the gradient computation. While the orbital rotation problem is typically resolved by increasing the active space size, here we keep the small active space consisting of only three orbitals, as only very few trajectories are affected by this problem.

The local diabatization algorithm was used for the integration of the electronic TDSE,^53,54^ with an integration time step of 0.02 fs for the electronic degrees of freedom and 0.5 fs for the nuclear degrees of freedom. The Granucci–Persico energy-based decoherence correction with the standard 0.1 a.u. parameter was applied during the SH dynamics.^82^ Rescaling of the nuclear velocities after a hop was performed in the direction of the nuclear momentum. The SH trajectories were propagated up to 150 fs but, in order to save computational time, were stopped if reached the electronic ground state and stayed there for at least 20 fs.

#### 3.1.2 Simulation of the excited-state dynamics

The simulated absorption spectrum, shown in Fig. 3, exhibits two peaks: a weaker one at lower energy corresponding to the forbidden nπ* excitations, and a stronger peak at higher energies corresponding to the ππ* excitation. The maximum of the nπ* state band (∼5.7 eV) qualitatively reproduces the vertical excitation obtained with previous results, *e.g.* with spin–flip time-dependent density functional theory (SF-TDDFT) (5.65 eV),^77^ linear-response time-dependent density functional theory (LR-TDDFT) (4.84 eV and 4.92 eV),^76,79^ configuration interaction (∼5 eV),^83^ quantum Monte Carlo (5.32 eV),^84^ and the experiment (∼4.96 eV).^85^

HN=CH_2_, the smallest unprotonated Schiff base, is a prototypical system for studying *cis*–*trans* photoisomerization around a double bond. The photophysical properties and associated nonadiabatic photoisomerization mechanism for this system have been rigorously studied using nonadiabatic *ab initio* molecular dynamics with LR-TDDFT,^76^ SH dynamics with semiempirical potentials and SF-TDDFT,^77,78^ and high-level “static” multireference configuration interaction calculations.^83^ In the electronic ground state, S_0_, HN=CH_2_ adopts the planar geometry depicted in Fig. 2. The system contains five atoms and therefore nine internal degrees of freedom that are all free to relax during the excited state dynamics simulations. After vertical excitation to the lowest excited singlet state, S_1_, it is known that the system rapidly relaxes toward the local energy minimum on the excited state PES and the N–H bond vector twists out of the molecular plane.^76–78^ On approaching the orthogonal twist geometry, the system enters the conical intersection region, resulting in strong nonadiabatic coupling between the S_1_ and S_0_ states. After relaxation to the S_0_ state, HN=CH_2_ returns to a planar geometry, leading either to the photoisomerized product or to the regeneration of the reactant state.

**Fig. 4:**
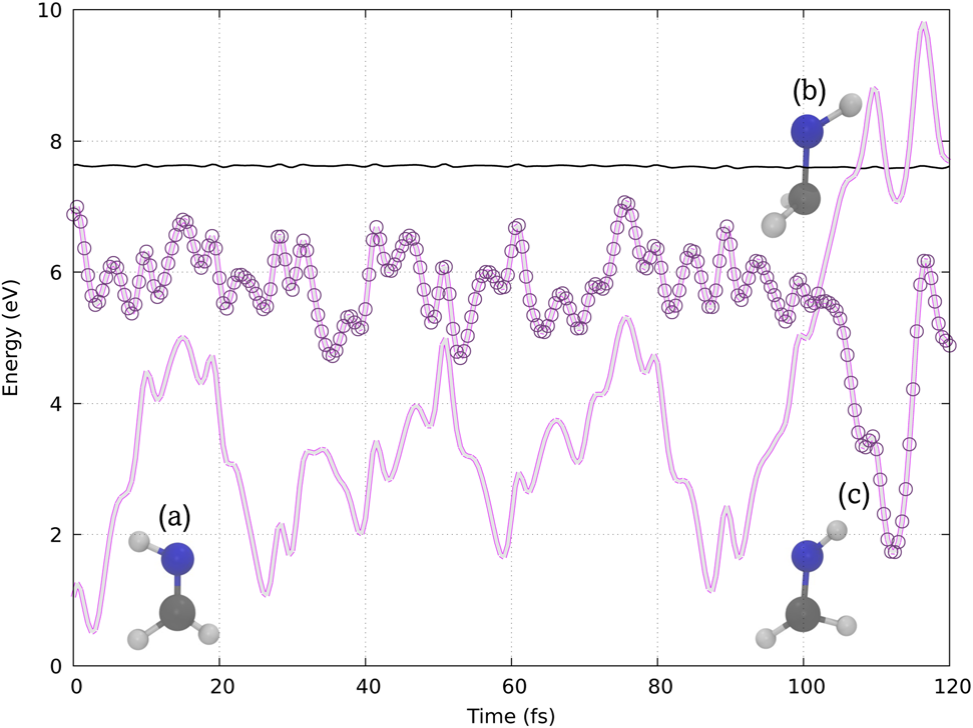
Potential energy surfaces calculated with VQE/VQD for a typical reactive *cis*–*trans* trajectory of HN=CH_2_, illustrating (a) the initial planar configuration, (b) the hopping structure with the N–H bond vector twisted out of the plane, and (c) the photoisomerized structure. The thin black line below 8 eV represents the total energy (kinetic + potential) over time and the circles represent the active adiabatic state.

Our simulations reproduce a similar behavior, as shown by the example trajectory in Fig. 4. Initially, in structure (a), the molecule presents a planar configuration. Structure (b) represents the hopping structure, where the N–H bond vector twists out of the molecular plane, followed by photoisomerization, leading to structure (c) in one of the last time steps. Other trajectories are shown in Section S3 (ESI).†

**Fig. 5:**
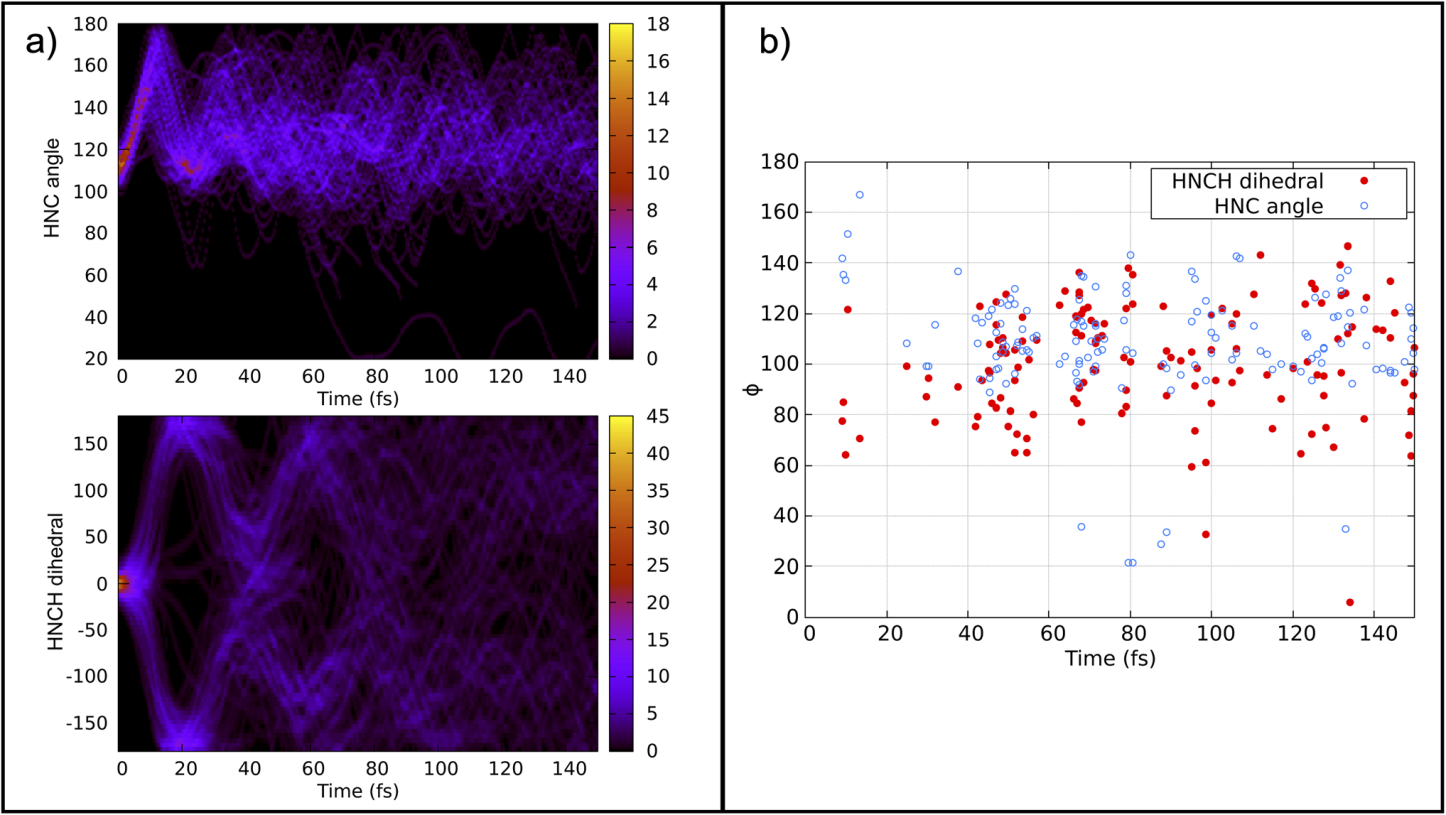
Panel (a) top: convolution of the HNC angle over time from 141 trajectories. Panel (a) bottom: convolution of the HNCH dihedral (dihedral among the atoms 1–2–3–4; see Fig. 2) over time from all trajectories. Panel (b): HNCH dihedral (dihedral among the atoms 1–2–3–4; see Fig. 2), in red, and HCN angle, in blue, at the S_1_–S_0_/S_0_–S_1_ hopping moment.

Previous calculations using classical algorithms reported that the crossing point is characterized by an HNC angle and an HNCH dihedral of approximately 100°. For instance, Tavernelli *et al.* reported an HNC angle and an HNCH dihedral of ∼100°,^76^ while Bonačić-Koutecký and Michl reported an HNC angle of ∼106.5° at the crossing point.^86^ We calculated the average of these internal coordinates for all the hopping geometries between the S_0_ and S_1_ states. Our findings show an average HNC angle of 108.4° and an HNCH dihedral (dihedral among the atoms 1–2–3–4; see Fig. 2) of 100.5°, which is in very good agreement with previous studies carried out on purely classical computers. In Fig. 5 panel (b), we show the HNC angles and the HNCH dihedral at the hopping geometries between S_0_–S_1_ and S_1_–S_2_. We note that most of the hops take place when both angles and dihedrals are in the range of 80–120°. This confirms that our approach can accurately reproduce the conical intersection geometries and is stable even at strong nonadiabatic coupling regions.

Fig. 5 panel (a) depicts the convolution of the HNC angle and the HNCH dihedral over time from all the trajectories considered. The HNC oscillates from 100–180° with a periodicity of 20 fs, while the HNCH dihedral oscillates from 0–180° with a periodicity of 40 fs. This periodicity diminishes as the trajectories start to decay to the ground state. Note that the strong coupling regime is achieved mainly when both internal coordinates are close to 100°.

**Fig. 6:**
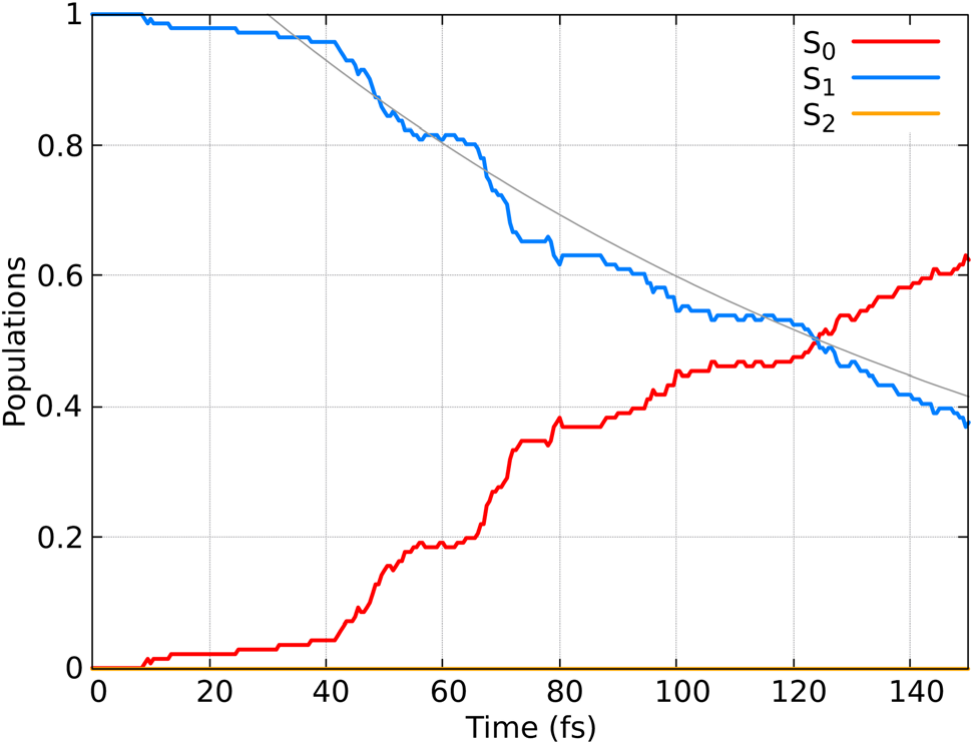
Time-resolved adiabatic state populations of HN=CH_2_, averaged over 141 trajectories. The gray line represents the fit of the S_1_ population according to the kinetic model described by eqn (21).

In Fig. 6 the population dynamics of the adiabatic states averaged over all trajectories following nπ* excitation is shown. Initially, all populations are in the S_1_ state, which has nπ* character, while the S_2_ (ππ*) state remains unpopulated throughout the dynamics. This observation aligns with the energy gap between these states (see Fig. 3). From the beginning of the dynamics, until approximately 30–40 fs, only a few trajectories decay to the ground state S_0_. Subsequently, an exponential decay to the ground state is observed.

To quantify this decay, we fit the S_1_ population *P*_S_1__ using a simple exponential decay model with a delay time *t*_0_. This model is described by the equation:21
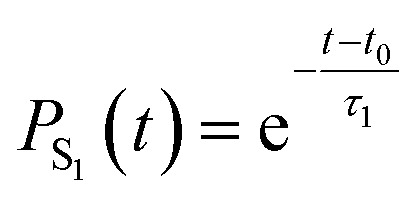
where *τ*_1_ is the decay constant, and the sum *τ*_1_ + *t*_0_ gives the lifetime of the S_1_ population. Here, we used a delay time *t*_0_ = 30.00 fs, which resulted in a decay constant *τ*_1_ = 136 fs. Therefore, the lifetime of the S_1_ population is 167 fs.

The observed population decay behavior is consistent with previous findings, *e.g.* those reported in ref. 78, where they used SH dynamics and semiempirical PESs, finding a similar “plateau” in the S_1_ population until 25–30 fs, followed by an exponential decay to the ground state. Dynamical simulations using SF-TDDFT^77^ showed a faster decay (∼58 fs) to the ground state compared to our observations. The differences can be attributed to minor variations in the PESs, that in return can have a substantial impact on the lifetimes. Additionally, the higher energy position of the nπ* band reported by us compared to the experimental spectrum^85^ and previous TDDFT calculations^77^ may contribute to the observed delay in the decay to the ground state.

Here, calculating the photoisomerization quantum yield is not straightforward because it can only be determined for trajectories that end in the ground state. However, we terminated the trajectories after 20 fs in the ground state, which is insufficient for the system to fully relax to the final product. As shown in Fig. 6, 62% of the population (corresponding to 88 trajectories) relaxed to the ground state by at the end of the propagation. Among these 88 trajectories, 30 exhibited an HNCH dihedral angle of ≤50°, suggesting that the final product is likely the non-photoisomerized form (we analysed the dihedral among the atoms 1–2–3–4; see Fig. 2), while 38 trajectories exhibited an HNCH dihedral angle ≥125°, indicating that the product most likely remains in the photoisomerized form. The remaining 20 trajectories, which also ended in the ground state, had dihedral angles between 50–125°, so we did not assign a final product to them. Considering only the 68 trajectories with definitive outcomes (30 *cis* and 38 *trans*), we estimate a “partial” photoisomerization quantum yield, *Φ*, of approximately 56% with a standard deviation of ±6%. This standard deviation is calculated according to the binomial standard deviation formula: 
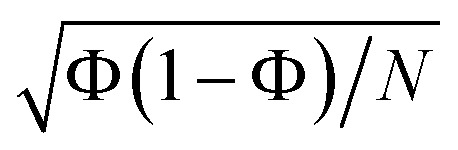
. This result is consistent with the results obtained for SH dynamics using semiempirical PES (54%).^78^ However, it is lower than the results from nonadiabatic Car–Parrinello molecular dynamics, which reported a value of 69%.^80^

Finally, we investigated whether the VQD algorithm can always find states that are orthogonal to each other. Therefore, we calculated the overlap between the electronic states 〈*Ψ*(*θ*_*m*_)|*Ψ*(*θ*_*l*_)〉 over time. Note that this overlap is not the same as in eqn (19). That overlap pertains to states at different times, meaning they represent different sets of molecular orbitals and its computation is more sophisticated. In Section S2 (ESI†), we shows for four different trajectories, how the overlaps between states S_0_–S_1_, S_0_–S_2_, and S_1_–S_2_ evolve over time. We observe that the overlaps are generally very small, indicating that most of the time, the VQD converges to states that are orthogonal to each other. In a few instances during the dynamics, the overlap can reach values of the order of 10^−3^. However, these overlaps always involve the S_2_ state, which does not participate in the dynamics. The overlaps between S_0_–S_1_, which are the states participating in the dynamics, remain very small, generally bellow to 1 × 10^−5^. From this, we conclude that the VQD is robust enough to reproduce the correct dynamical behaviour during the propagation.

### 3.2 Test case CH_2_=CH_2_

#### 3.2.1 Computational details

A similar approach is used to perform the SH dynamics with slight differences.

The VQE/VQD electronic energies and wavefunctions were also obtained using an active space of 4 electrons and 3 orbitals (6 spin–orbitals and 6 qubits), which involves one π, one σ, and one π*. A HF/6-31G wavefunction was used as a reference, calculated with PySCF. The Jordan–Wigner mapping was applied to generate the qubit Hamiltonian, and both VQE and VQD used the BFGS optimizer to minimize the parameters *θ*. In the first time step, the parameters *θ* used were the optimal ones for HN=CH_2_. In attempting to set them to zero, as we did at the first time step of HN=CH_2_, the VQD did not find the state corresponding to the ππ* transitions (this state can be the S_1_ or the S_2_ state depending on the geometry). However, for subsequent time steps, the initial *θ* values were those obtained from the optimization of the previous time step. For the first excited state, 6 angles were optimized, resulting in an average of 7.45 iterations per nuclear time step over a single trajectory (with a time step of 0.5 fs). In the case of the second excited state, 13 angles were utilized, but only 7 were optimized, which required an average of 23.45 iterations. For the third excited state, 19 angles were involved, with 6 optimized, averaging 26.25 iterations per nuclear time step.

**Fig. 7:**
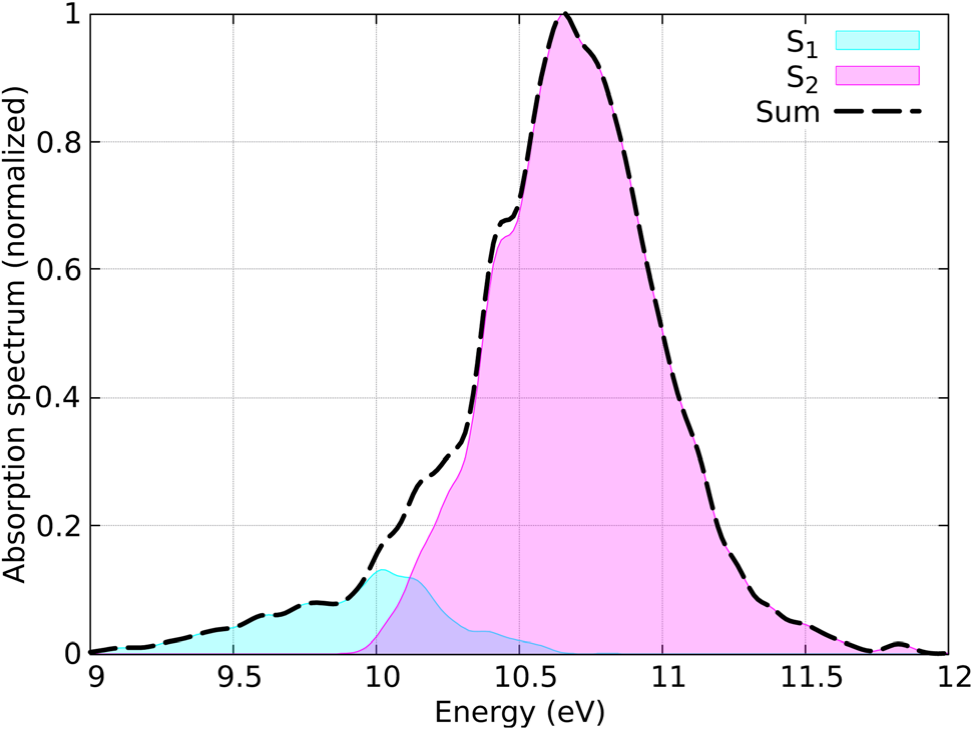
Simulated absorption spectrum of CH_2_=CH_2_ calculated using 5000 geometries. Contributions from each adiabatic state are indicated, with the dashed line representing the total spectrum.

The distribution of coordinates and velocities was also generated by a Wigner distribution and the corresponding spectrum is shown in Fig. 7. The starting conditions (geometries, velocities, and initial state) were selected according to the excitation window ranging from 10.5 eV to 11.0 eV, which corresponds to the excitation to the S_2_ state (ππ* excitation). A total of 100 initial conditions were extracted from a nuclear ensemble of 5000 geometries. In total, 85 were used to analyze the dynamics; 15 trajectories were discarded because the total energy was not conserved by the end of the dynamics. This energy inconsistency is primarily due to orbital rotation within the active space, resulting in discontinuities in the PESs and thus in violation in the total energy conservation. Capturing all different processes occurring in ethylene upon light irradiation, also including dissociation of hydrogen atoms, is a notorious-complicated problem that requires a large active space.^87^ For the sake of practicality, we keep our small active space and removed the problematic trajectories. In the SH simulations, we used the local diabatization algorithm for the integration of the electronic TDSE, with an integration time step of 0.02 fs for the electronic degrees of freedom and 0.5 fs for the nuclear degrees of freedom. For some trajectories where the total energy was not conserved, the nuclear time step was reduced to 0.25 fs. The Granucci and Persico energy-based decoherence correction with the standard 0.1 a.u. parameter was applied during the SH dynamics. The rescaling of the nuclear velocities after a hop was performed in the direction of the nuclear momentum. The SH trajectories were propagated up to 100 fs; however, the trajectories that reached the ground state were stopped after running on the ground state for at least 10 fs.

#### 3.2.2 Simulation of the excited states dynamics

The simulated absorption spectrum, shown in Fig. 7 exhibits one peak with two contributions. They come from the S_1_ and S_2_ states, even if they both correspond to the ππ* transition, which for different initial geometries is located at one of the S_1_ or S_2_ states. Most of the time, for the geometries picked by the Wigner distribution, the S_1_ state corresponds to the σπ* excitation, which does not contribute to the spectrum. We note that the S_1_ and S_2_ states are very close in energy.

CH_2_=CH_2_ contains six atoms and therefore has twelve internal degrees of freedom that are all free to relax during the excited state dynamics simulations. Its compact structure, extremely fast dynamics (under 100 fs) through a conical intersection, and significant conformational flexibility have made this molecule a frequently used system for benchmarks and testing method developments, including mixed-quantum classical dynamics methods.^62–75,88^

Previous SH-based studies of CH_2_=CH_2_ have discussed its dynamics in detail.^62,63,69,70,73^ In essence, after the ππ* state is excited, it decays very rapidly to the ground state. In doing so, CH_2_=CH_2_ transfers strong intramolecular vibrational redistribution of energy takes place, and besides photoisomerizing, it can show photodissociation. For example, it can release one or two H atoms and ethylidene isomer (CH_3_–CH) is also a common photoproduct. Yet, CH_2_=CH_2_ is still the most common structure.^63^ Several static studies and dynamics reveal that the most common conical intersection structure presents a twisted-pyramidalized geometry.^89–91^

In our analysis, we do not focus on identifying and quantifying the photoproducts, as we terminate the dynamics after 10 fs in the electronic ground state.

**Fig. 8:**
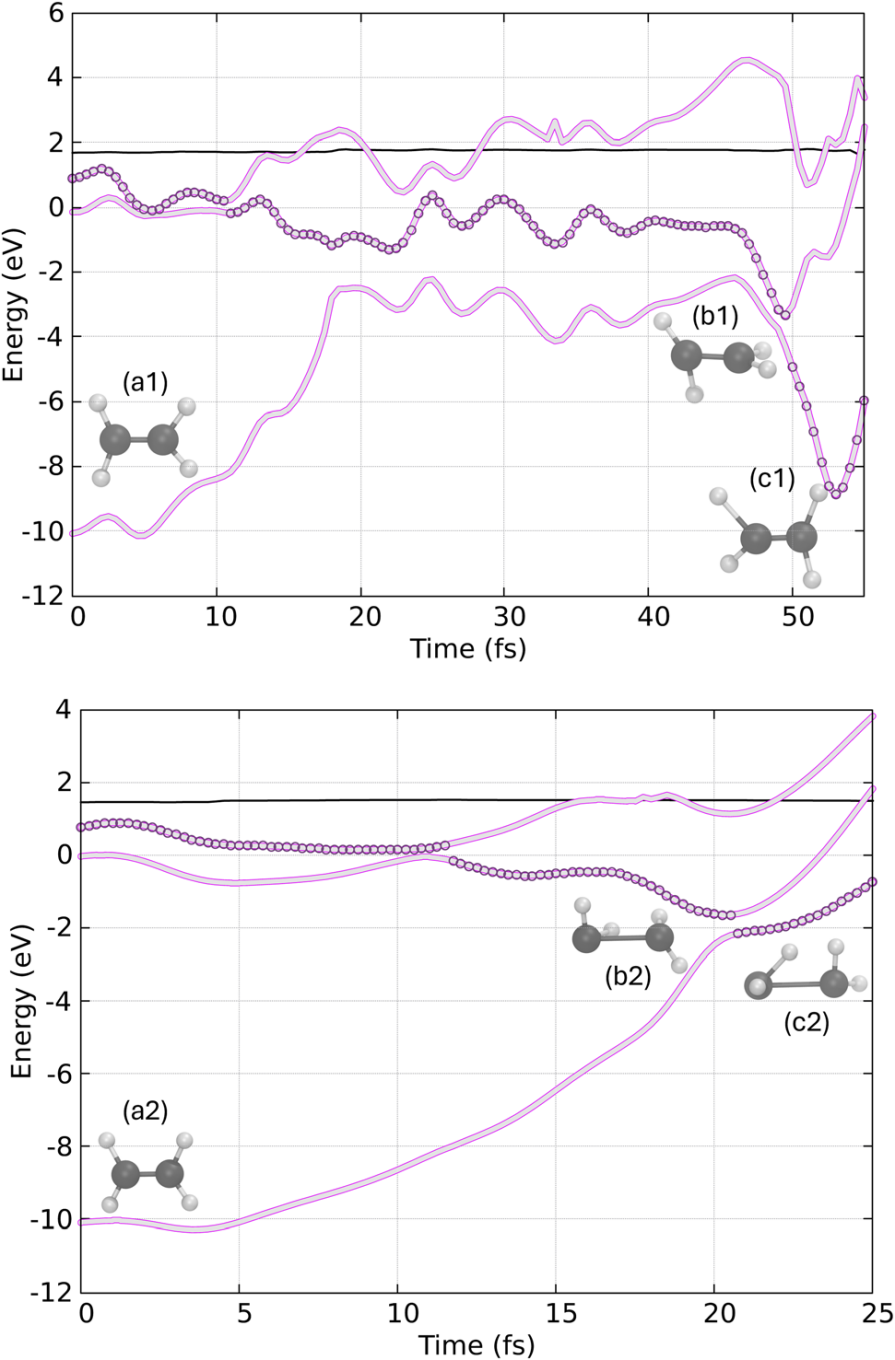
Potential energy surfaces calculated with VQE/VQD for two trajectories of CH_2_=CH_2_, illustrating (a1) and (a2) the initial configurations, (b1) and (b2) the hopping structure and (c1) and (c2) the final geometry. The thin black line below 2 eV represents the total energy (kinetic + potential) over time and the circles represent the active adiabatic state.

In Fig. 8, we present two example trajectories. In the first trajectory (top), the S_1_ state is populated just before 10 fs, and by 50 fs, the system undergoes a hop to the ground state. The hopping structure (geometry b1) exhibits a twisted-pyramidalized geometry, closely resembling the conical intersection geometry reported in previous static and dynamic studies.^62–75,88–91^ Consequently, the final geometry at the end of the dynamics (geometry c1) suggests that the final product is CH_2_=CH_2_.

In the second trajectory (bottom of Fig. 8), we observe the decay of the S_2_ state to the S_1_ state shortly after 10 fs, followed by a rapid decay to the ground state just after 20 fs. In this case, the hopping structure (geometry b2) also displays a twisted-pyramidalized geometry. However, the final geometry appears to be in the process of forming the ethylidene isomer, as indicated by the apparent migration of a hydrogen atom to the other carbon.

**Fig. 9:**
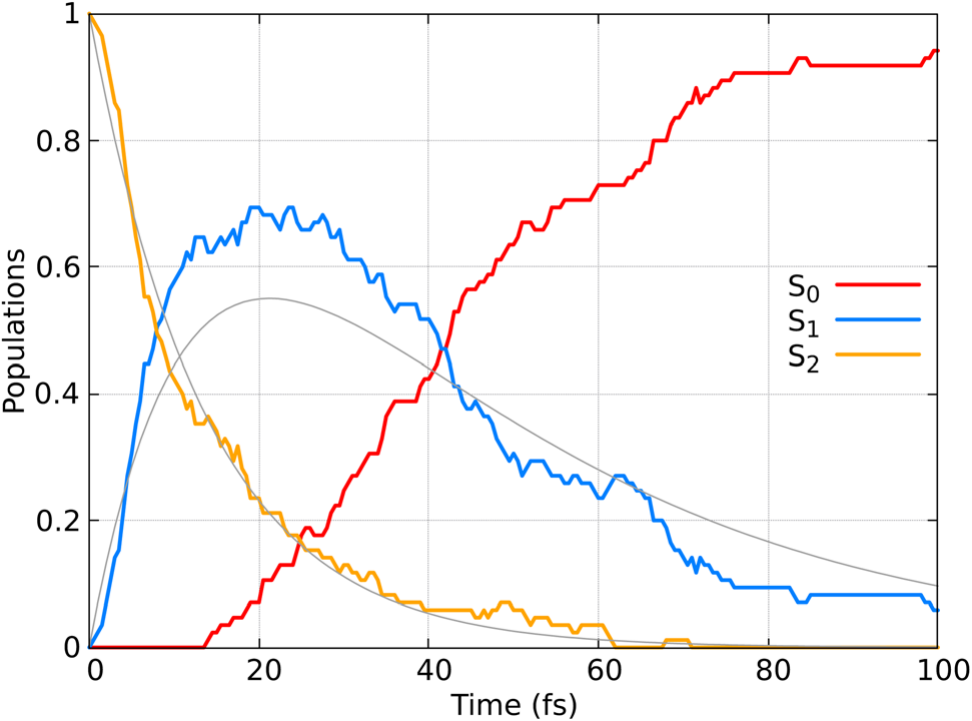
Adiabatic state populations as functions of time, averaged over 85 trajectories for CH_2_=CH_2_. The gray line represents the fit of the S_1_ and S_2_ populations according to the kinetic model described by eqn (22).

Fig. 9 displays the population dynamics of the adiabatic states averaged over all trajectories following ππ* excitation. Initially, all populations reside in the S_2_ state, characterized by ππ* character. The S_2_ state undergoes a very fast exponential decay to the S_1_ state which decays rapidly to the ground state. It is crucial to emphasize that in our dynamics, the σπ* state is never populated. Instead, the ππ* state, which initially corresponds to the S_2_ and later becomes the S_1_ state, decays to the ground state.

Previous SH dynamics studies also reported that the initially populated ππ* state is responsible for decaying to the ground state.^62,63,69,70^

To quantify the population decay, we fit a two-step irreversible kinetics model, defining the lifetimes *τ*_1_ and *τ*_2_ of the respective excited state manifolds as22
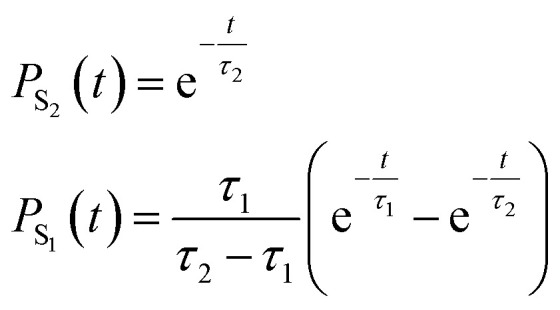
where *τ*_2_ is the lifetime of the S_2_ population (*P*_S_2__), yielding *τ*_2_ = 14 fs, and *τ*_1_ is the lifetime of the S_1_ population (*P*_S_1__), yielding *τ*_1_ = 35 fs. This ultrafast decay of ∼50 fs (*τ*_1_ + *τ*_2_) aligns well with experimental results, where time-resolved spectroscopy has shown that ethylene's internal conversion occurs in only 10–30 fs.^92–96^ We note that our obtained lifetime is also in agreement with other nonadiabatic dynamics studies performed for CH_2_=CH_2_ on classical computers.^62–75,88^

## 4 Conclusions and future perspectives

We have reported a theoretical and computational framework to perform nonadiabatic dynamics simulations using a hybrid quantum-classical algorithm. By harnessing the variational quantum eigensolver and variational quantum deflation algorithms, we are able to accurately calculate ground and excited state energies, gradients, as well as nonadiabatic coupling vectors and transition dipole moments, which can all be used to perform nonadiabatic molecular dynamics simulations.

The trajectories were simulated within the framework of the Tully's fewest switches approach, using a local diabatization scheme for the integration of the electronic coefficients, albeit our method is versatile enough to be easily integrated into other mixed quantum-classical dynamics approaches. The presented methodology is integrated into our SHARC program package by making use of the TEQUILA quantum computing framework to compute the electronic properties.

Our approach was tested on two polyatomic molecular systems: the *cis*–*trans* photoisomerization of methanimine and the electronic relaxation of ethylene. The results demonstrate qualitatively accurate molecular dynamics for both cases, successfully reproducing findings from other computational studies and experimental data.

In this study, we employed the k-UpCCGSD ansatz for computing the electronic properties, but other ansätze can be seamlessly integrated. Furthermore, we anticipate extending this approach to other excited-state properties, such as spin–orbit couplings, by exploiting the flexibility of the VQD algorithm, which can include extra penalty terms to track spin multiplicity.

We believe this study represents a significant step towards realizing the “quantum advantage” for practical applications of quantum computers for chemical simulations. A thorough analysis of the robustness of the proposed algorithms against circuit noise present in real quantum computers is crucial before proceeding to run the calculations on NISQ quantum devices.

## Data availability

All simulation results are included in the article and/or ESI.† The integration of the TEQUILA quantum computing framework into the SHARC program package will be available in an upcoming release of SHARC, which is published under the GPL license at https://github.com/sharc-md/.

## Author contributions

E. S. G. conceptualized the study, implemented the methodology, performed dynamic simulations, curated the data, and wrote the original draft. M. O. supervised the project, assisted in the conceptualization, and contributed to the review and editing of the manuscript. J. S. K. assisted in the implementation and conceptualization, and contributed to the review and editing of the manuscript. L. G. supervised the project, acquired funding, and contributed to the review and editing of the manuscript.

## Conflicts of interest

There are no conflicts to declare.

## Supplementary Material

SC-OLF-D4SC04987J-s001

SC-OLF-D4SC04987J-s002

SC-OLF-D4SC04987J-s003

SC-OLF-D4SC04987J-s004

SC-OLF-D4SC04987J-s005

SC-OLF-D4SC04987J-s006

SC-OLF-D4SC04987J-s007

SC-OLF-D4SC04987J-s008

SC-OLF-D4SC04987J-s009
