# Supplementary material for: SHARC meets TEQUILA: mixed quantum-classical dynamics on a quantum computer using a hybrid quantum-classical algorithm
Source: Chem Sci. 2024 Nov 28;16(2):596–609. doi: 10.1039/d4sc04987j (PMC11653199; doi:10.1039/d4sc04987j)
Supplement: SC-016-D4SC04987J-s001 [file SC-016-D4SC04987J-s001.pdf]

Supplementary Information (SI) for Chemical Science.
This journal is © The Royal Society of Chemistry 2024

SupplementaryInformation for:

"SHARC meets TEQUILA: Mixed Quantum-Classical Dynamics on a Quantum Computer using a Hybrid Quantum-Classical Algorithm"

Eduarda Sangiogo Gil,* Markus Oppel, Jakob S. Kottmann, and Leticia González†

Contents

S1 Gradients validation 1

S2 State overlap 4

S3 HN=CH_2 individual trajectories 5

S1 Gradients validation

In this section, we validate the computation of gradients by examining the significance of Pulay forces:

$$\langle \partial_{I,\xi}\Psi|\hat{\mathcal{H}}(\boldsymbol{R})|\Psi\rangle + \langle \Psi|\hat{\mathcal{H}}(\boldsymbol{R})|\partial_{I,\xi}\Psi\rangle.$$

For this, we compute the numerical differentiation of the electronic energies using a two-point finite difference formula with a step size of 0.001 Angstroms, and compare these with gradients that only consider the Hellmann-Feynman term, as shown in Eq. 6.

Our analysis focuses on the gradients of the S_0 and S_1 states of HN=CH_2. The geometries used in this analysis were extracted from three points in the trajectory: the beginning of the dynamics (Figs. S1 and S2), just before hopping to the ground state (Figs. S3 and S4), and at the end of the dynamics (Figs. S5 and S6).

The agreement between the approximate and exact gradients is satisfactory across all cases, with relatively small errors. Larger relative errors are observed only for small gradient values.

*eduarda.sangiogo.gil@univie.ac.at
†leticia.gonzalez@univie.ac.at

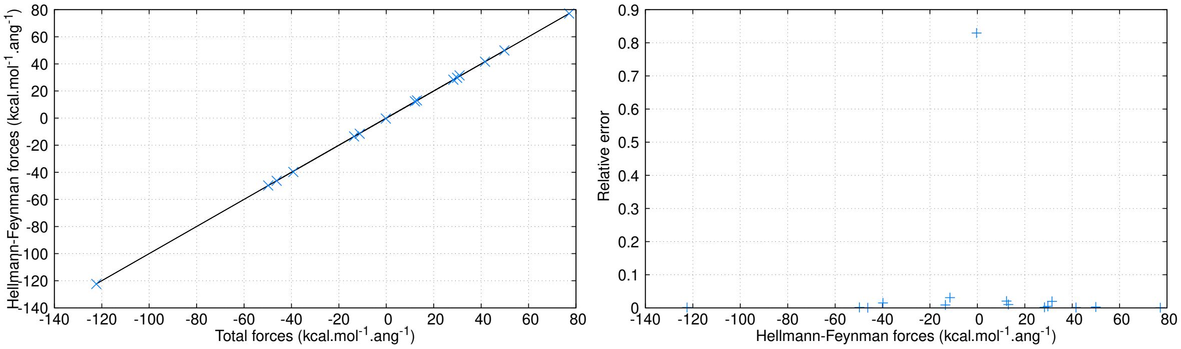

Figure S1: Left panel: Gradients of the S_0 state considering all contributions (Eq. 5) vs. gradients considering only the Hellmann-Feynman term for a geometry at the beginning of the dynamics. Right panel: Absolute value of the relative error of the approximate gradients.

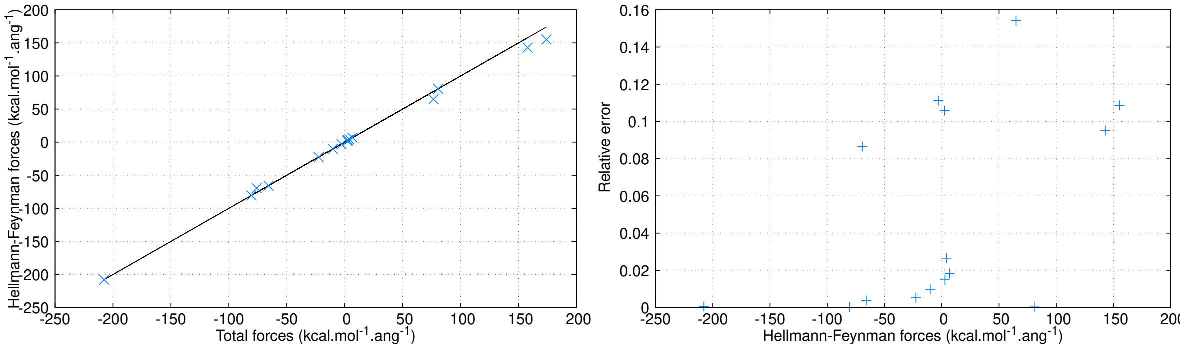

Figure S2: Left panel: Gradients of the S_1 state considering all contributions (Eq. 5) vs. gradients considering only the Hellmann-Feynman term for a geometry at the beginning of the dynamics. Right panel: Absolute value of the relative error of the approximate gradients.

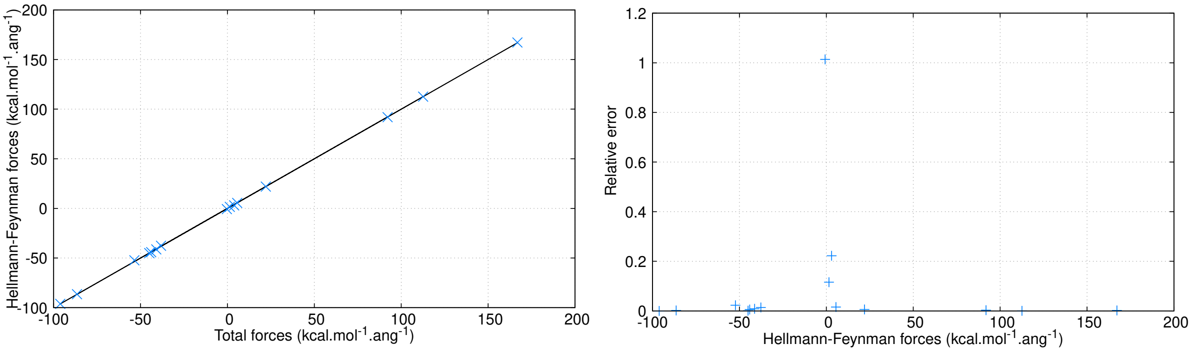

Figure S3: Left panel: Gradients of the S_0 state considering all contributions (Eq. 5) vs. gradients considering only the Hellmann-Feynman term for a geometry just before hopping to the ground state. Right panel: Absolute value of the relative error of the approximate gradients.

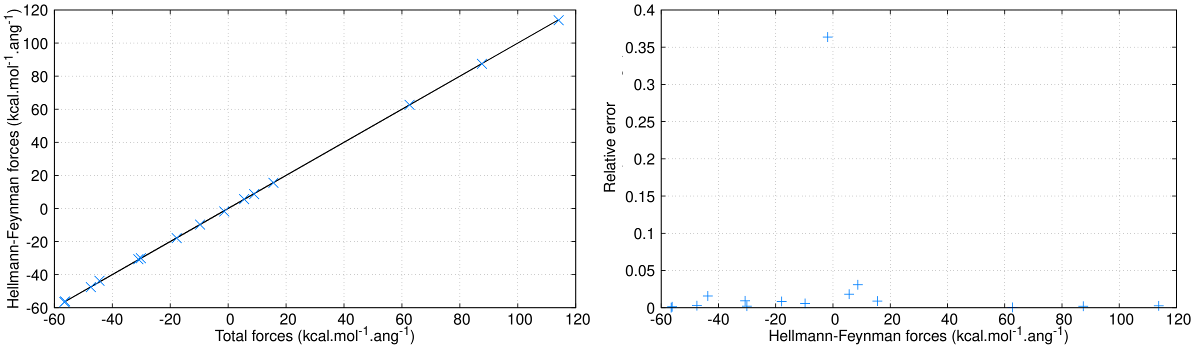

Figure S4: Left panel: Gradients of the S_1 state considering all contributions (Eq. 5) vs. gradients considering only the Hellmann-Feynman term for a geometry just before hopping to the ground state. Right panel: Absolute value of the relative error of the approximate gradients.

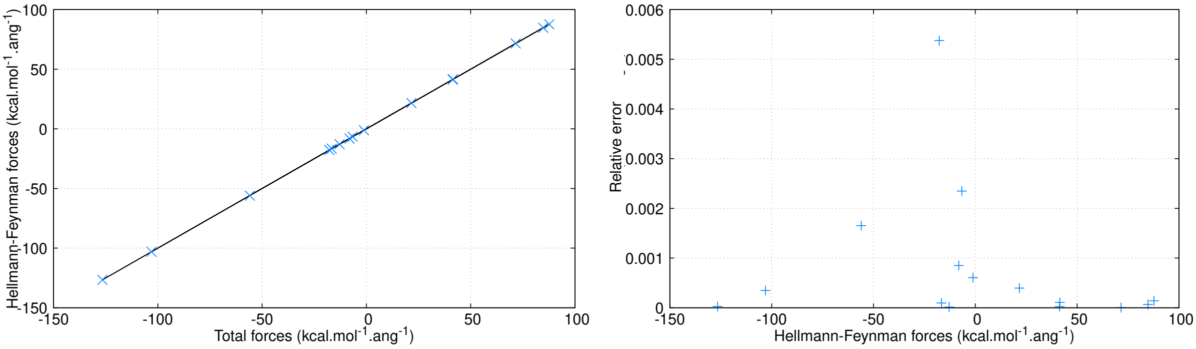

Figure S5: Left panel: Gradients of the S_0 state considering all contributions (Eq. 5) vs. gradients considering only the Hellmann-Feynman term for a geometry in the end of the dynamics. Right panel: Absolute value of the relative error of the approximate gradients.

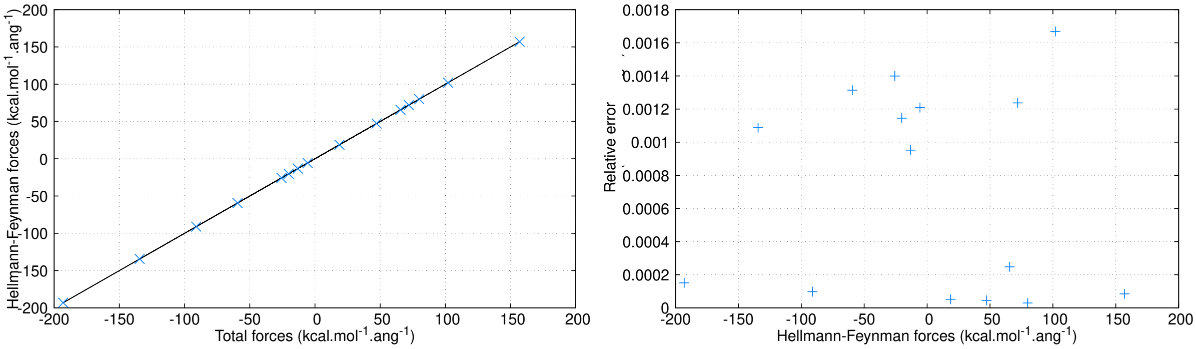

Figure S6: Left panel: Gradients of the S_1 state considering all contributions (Eq. 5) vs. gradients considering only the Hellmann-Feynman term for a geometry in the end of the dynamics. Right panel: Absolute value of the relative error of the approximate gradients.

S2 State overlap

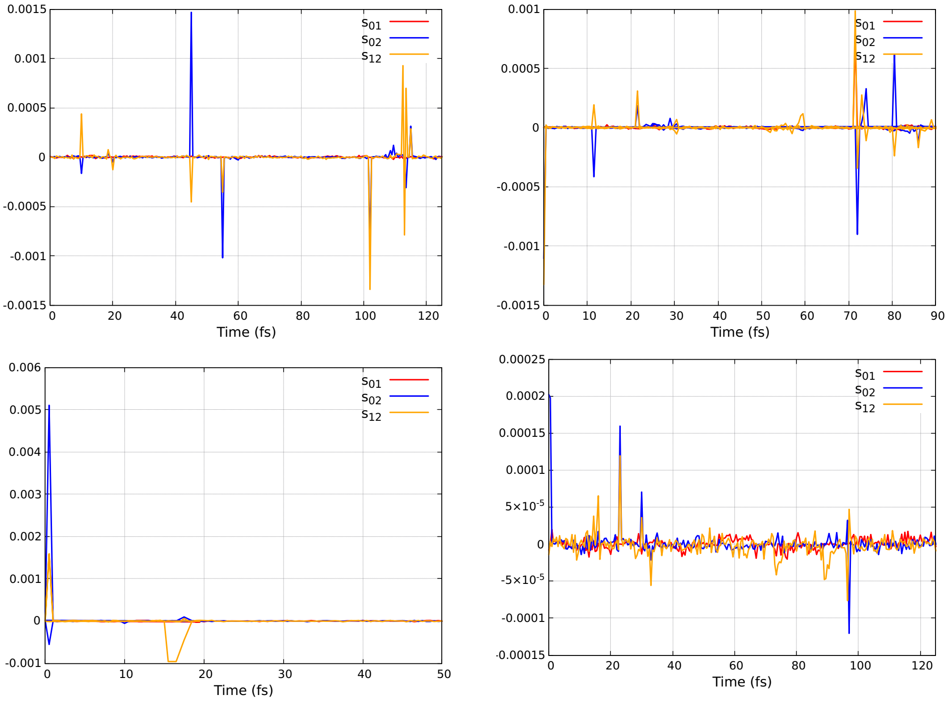

Figure S7: State overlap over time for four different trajectories. s_{01} is the overlap between states S_0 and S_1, s_{02} is the overlap between states S_0 and S_2, and s_{12} is the overlap between states S_1 and S_2.

S3 HN=CH_2 individual trajectories

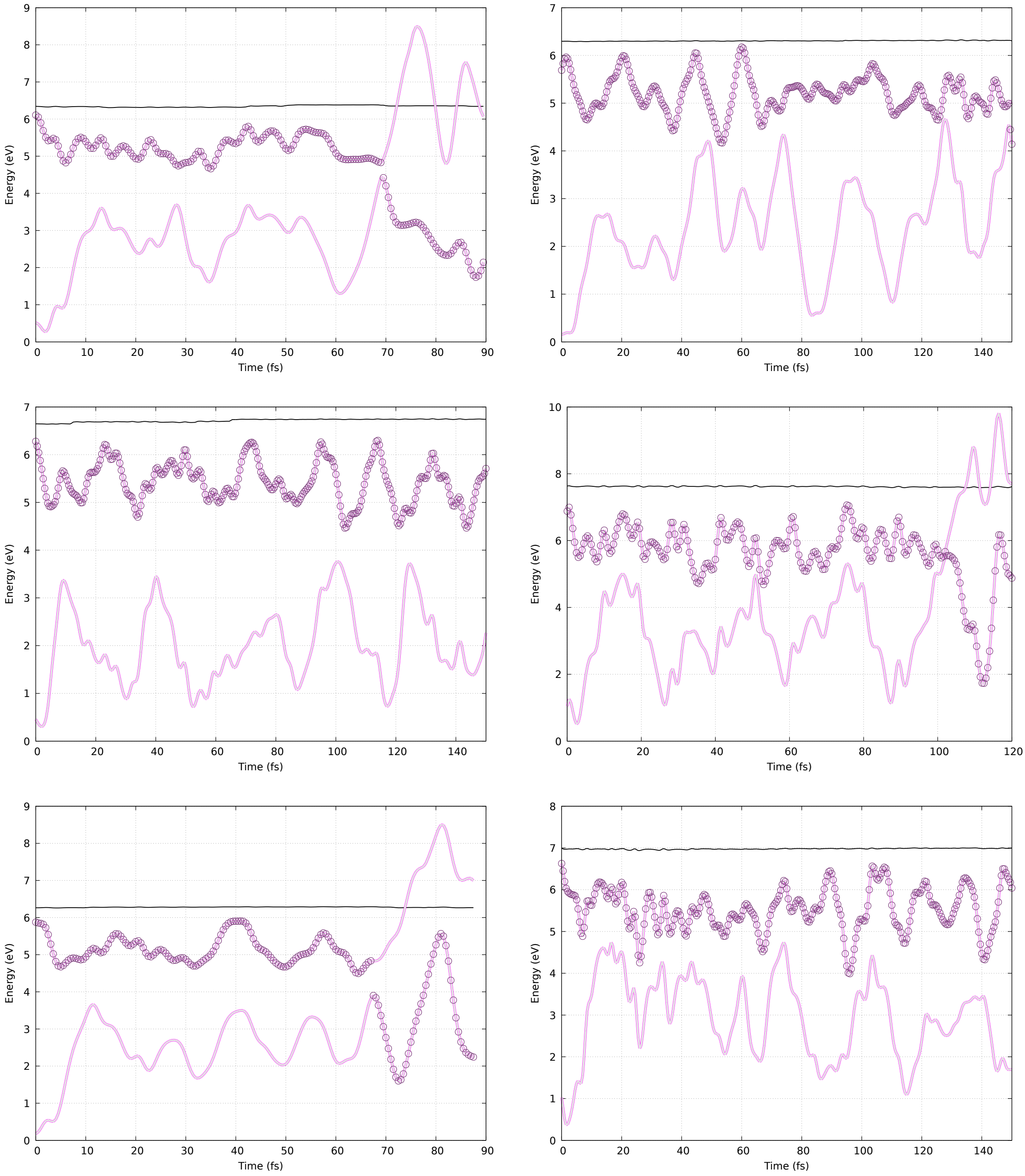

Figure S8: PES for six different trajectories of HN=CH_2. The thin black line represents the total energy (kinetic + potential) over time and the circles represent the active adiabatic state.
