# Supplementary figures and images for: SHARC meets TEQUILA: mixed quantum-classical dynamics on a quantum computer using a hybrid quantum-classical algorithm
Source: Chem Sci. 2024 Nov 28;16(2):596–609. doi: 10.1039/d4sc04987j (PMC11653199; doi:10.1039/d4sc04987j)

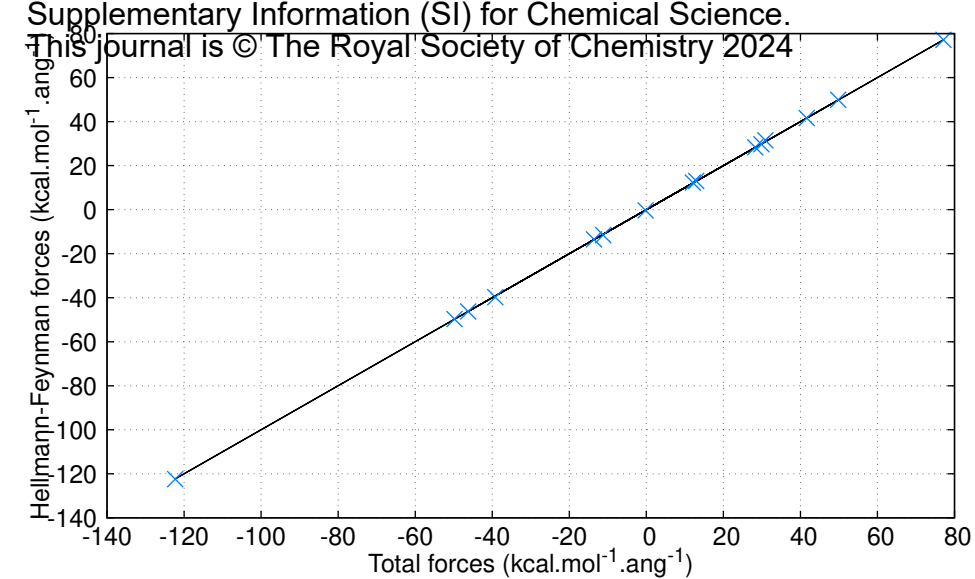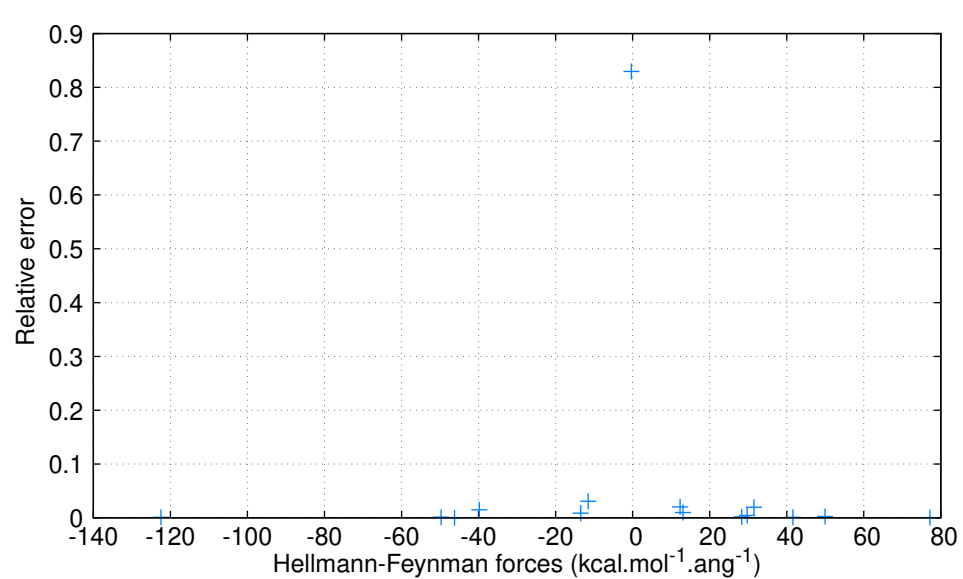

Supplement: SC-016-D4SC04987J-s002 [file SC-016-D4SC04987J-s002.pdf]

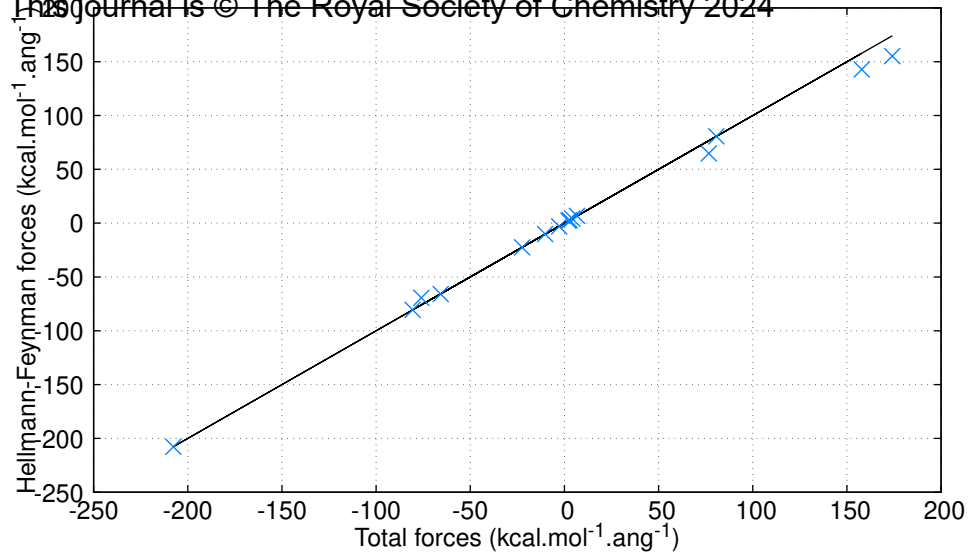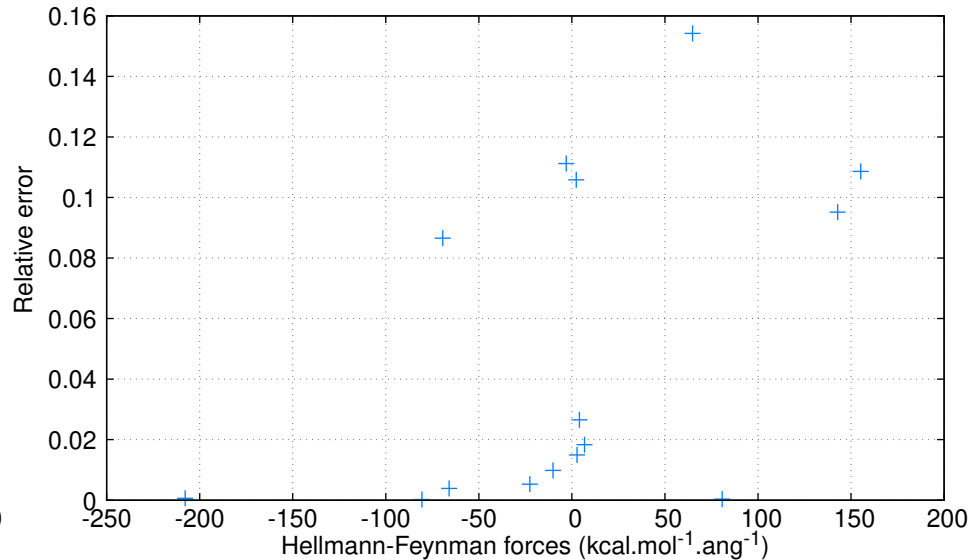

Supplement: SC-016-D4SC04987J-s003 [file SC-016-D4SC04987J-s003.pdf]

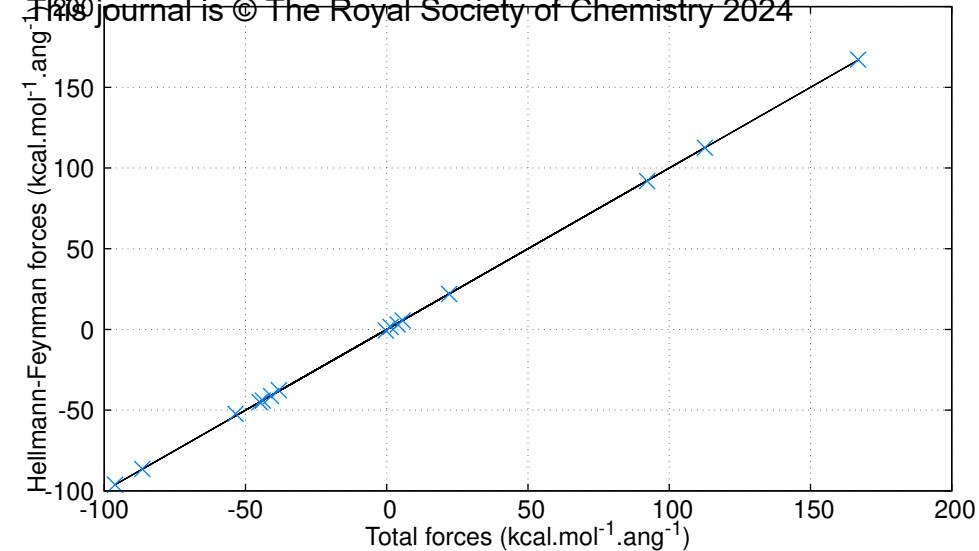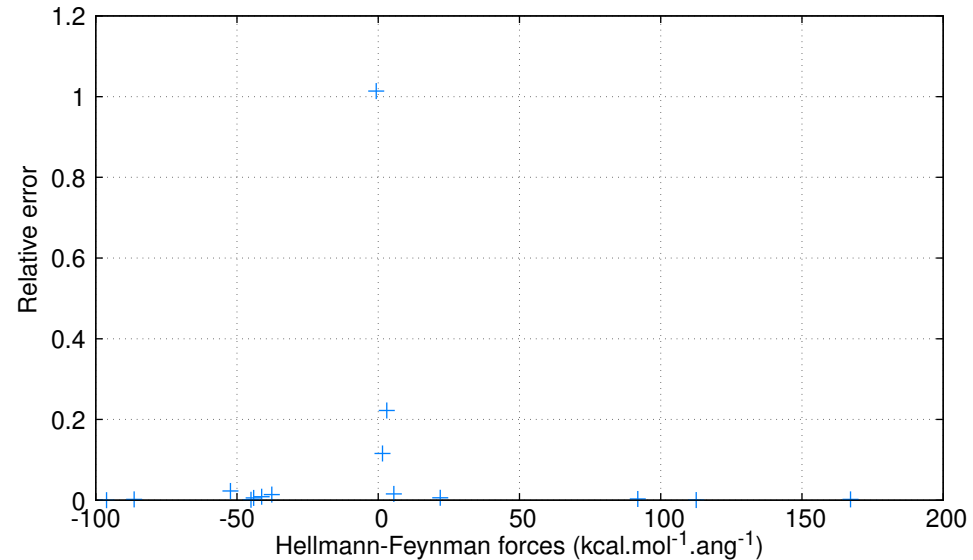

Supplement: SC-016-D4SC04987J-s004 [file SC-016-D4SC04987J-s004.pdf]

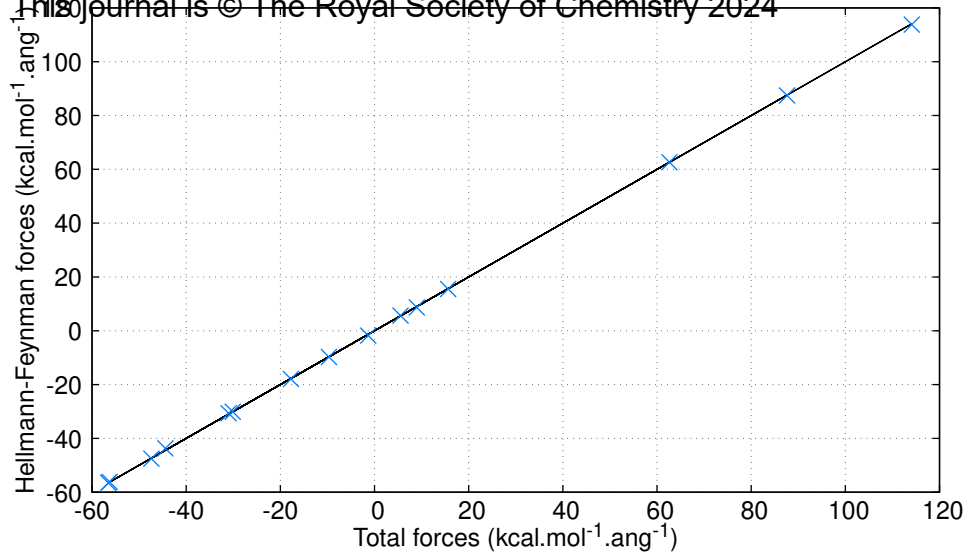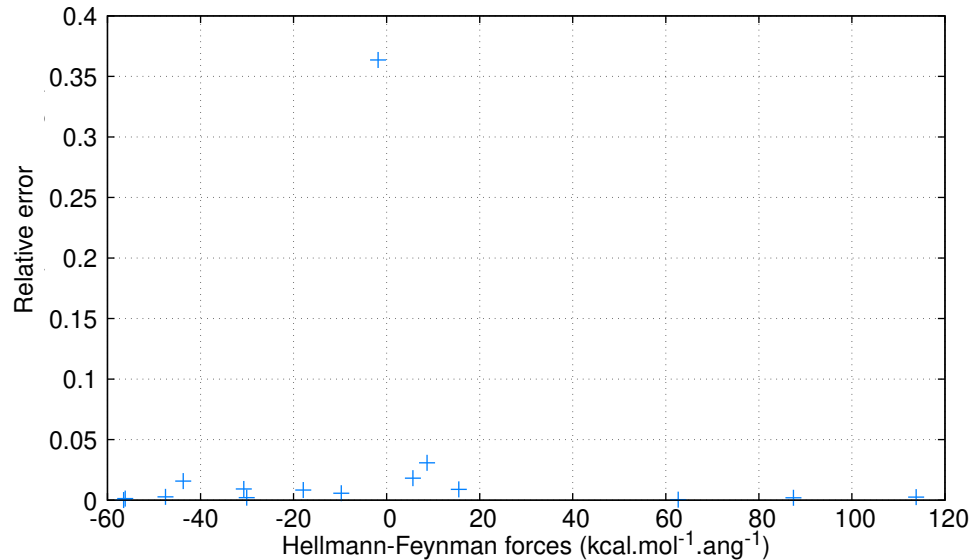

Supplement: SC-016-D4SC04987J-s005 [file SC-016-D4SC04987J-s005.pdf]

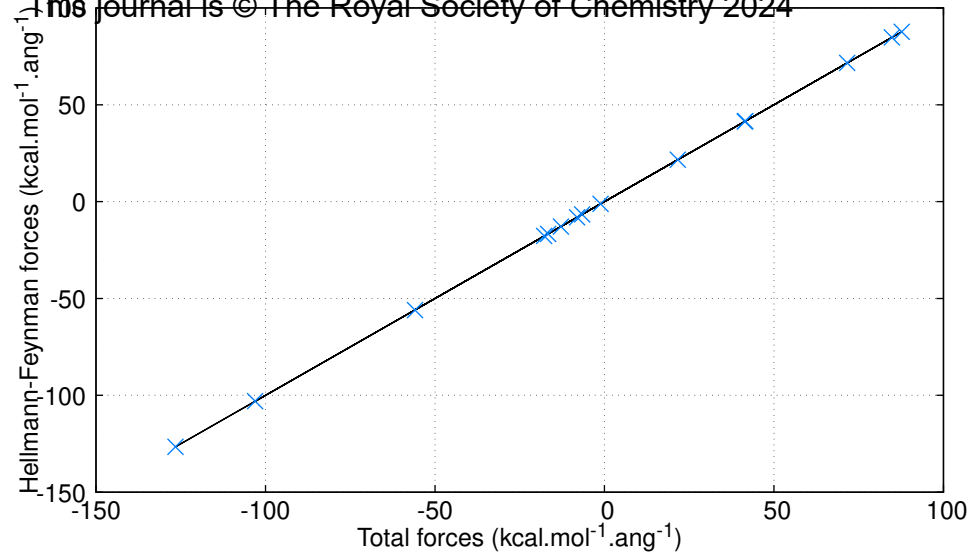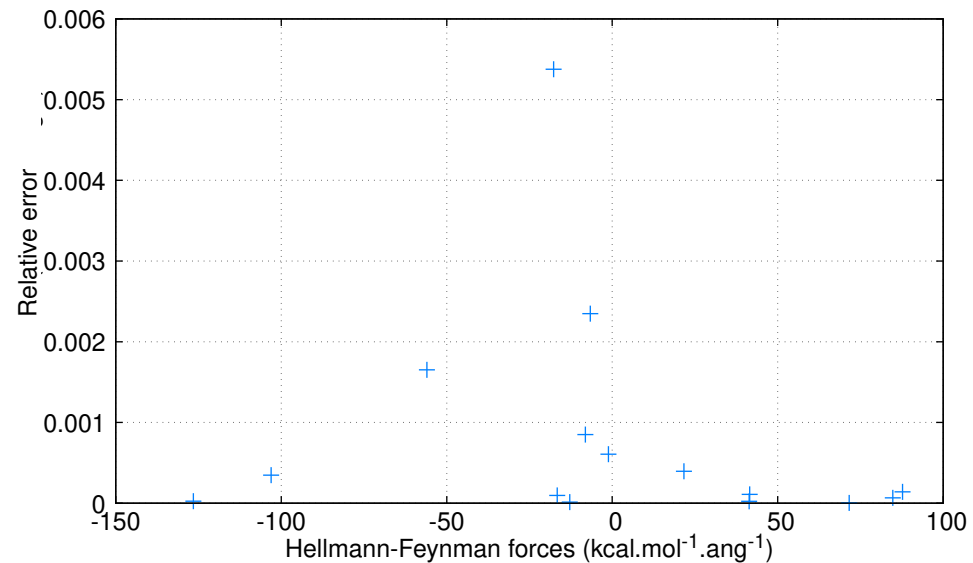

Supplement: SC-016-D4SC04987J-s006 [file SC-016-D4SC04987J-s006.pdf]

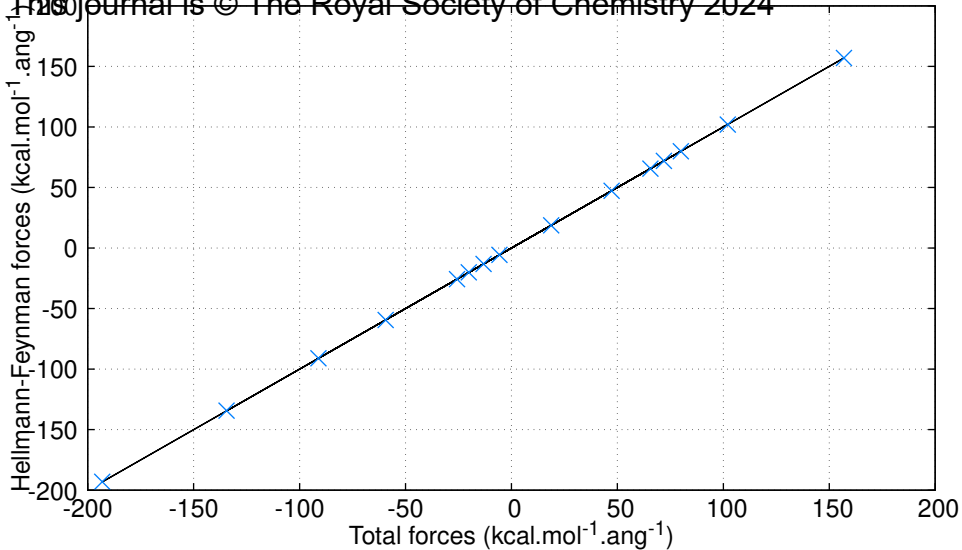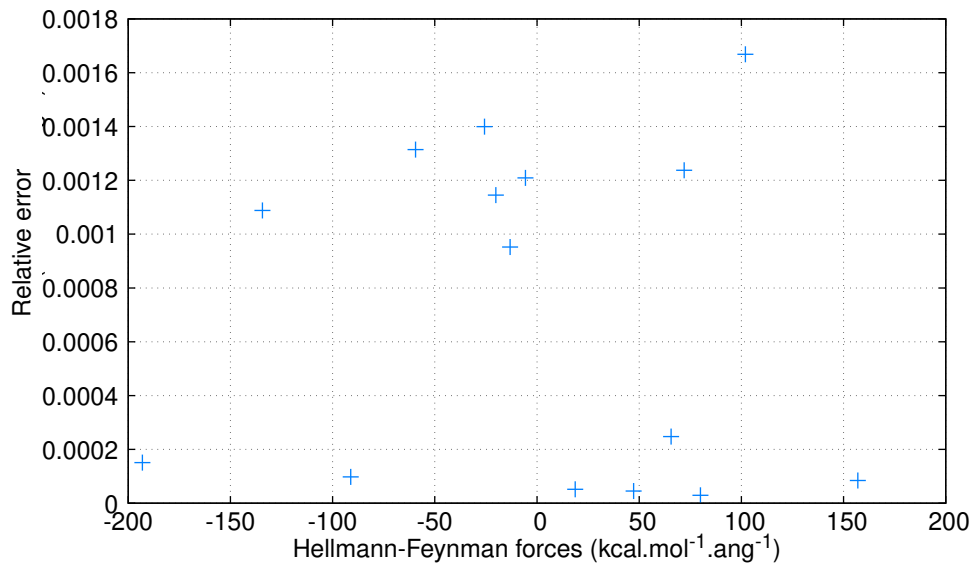

Supplement: SC-016-D4SC04987J-s007 [file SC-016-D4SC04987J-s007.pdf]

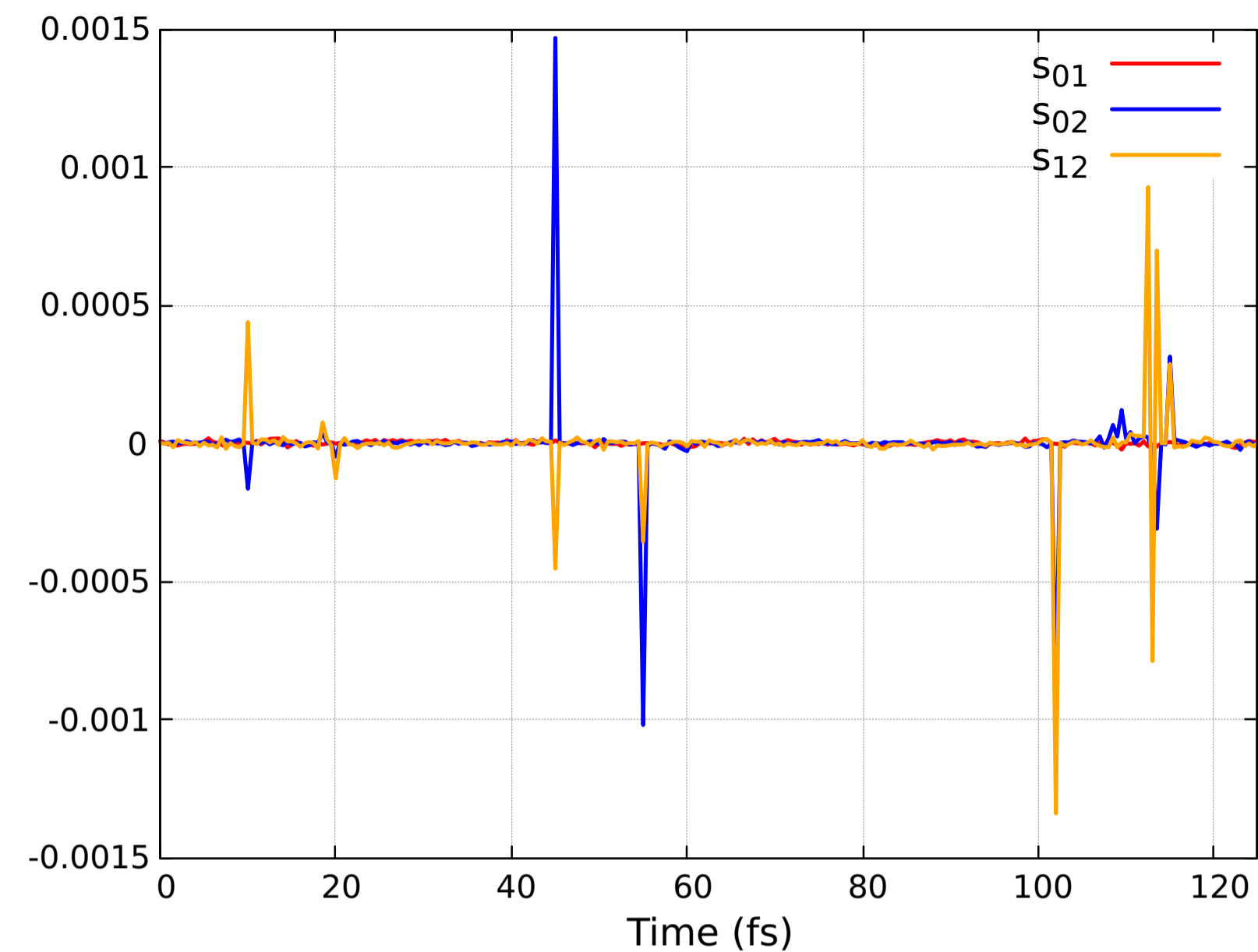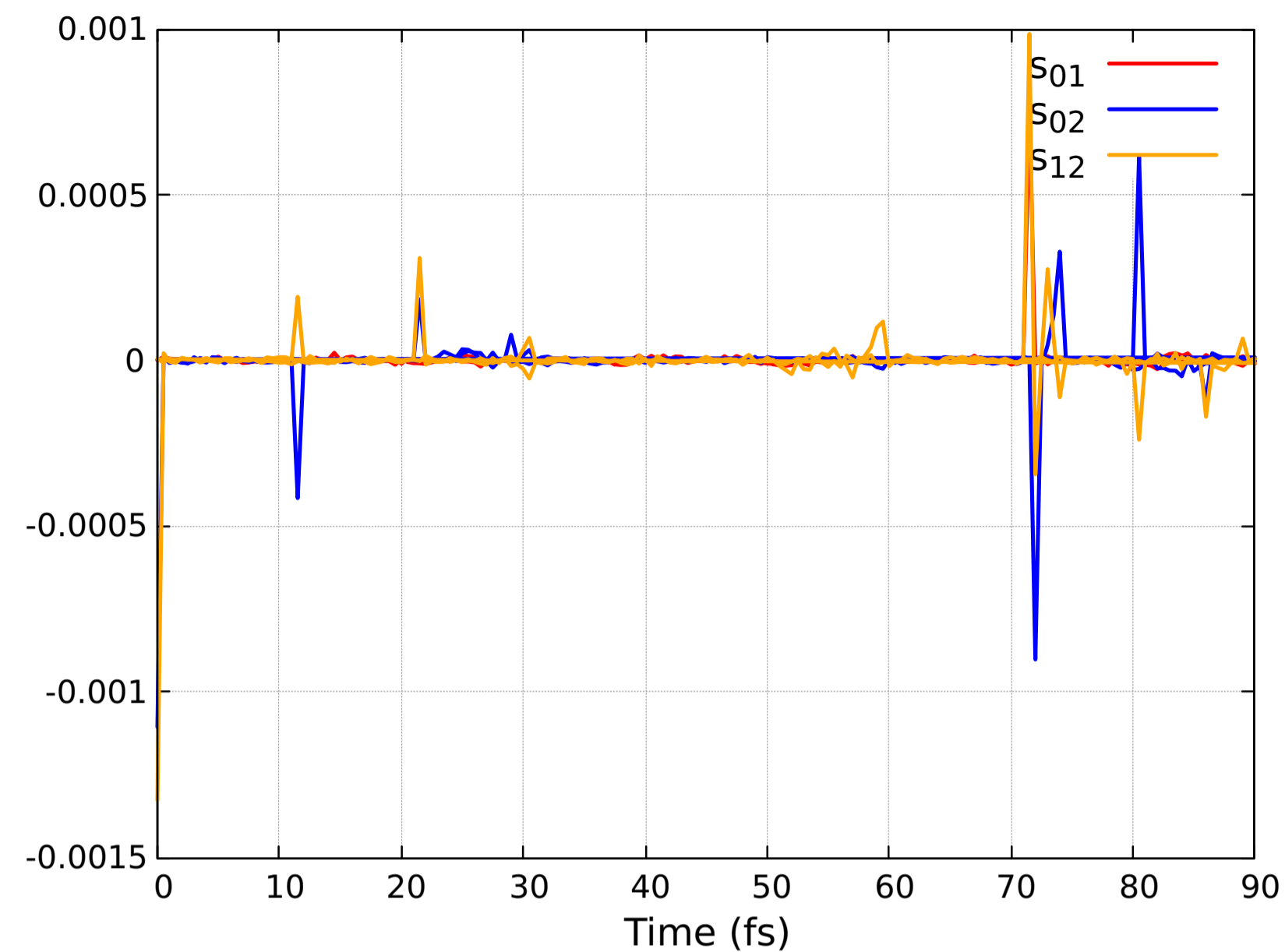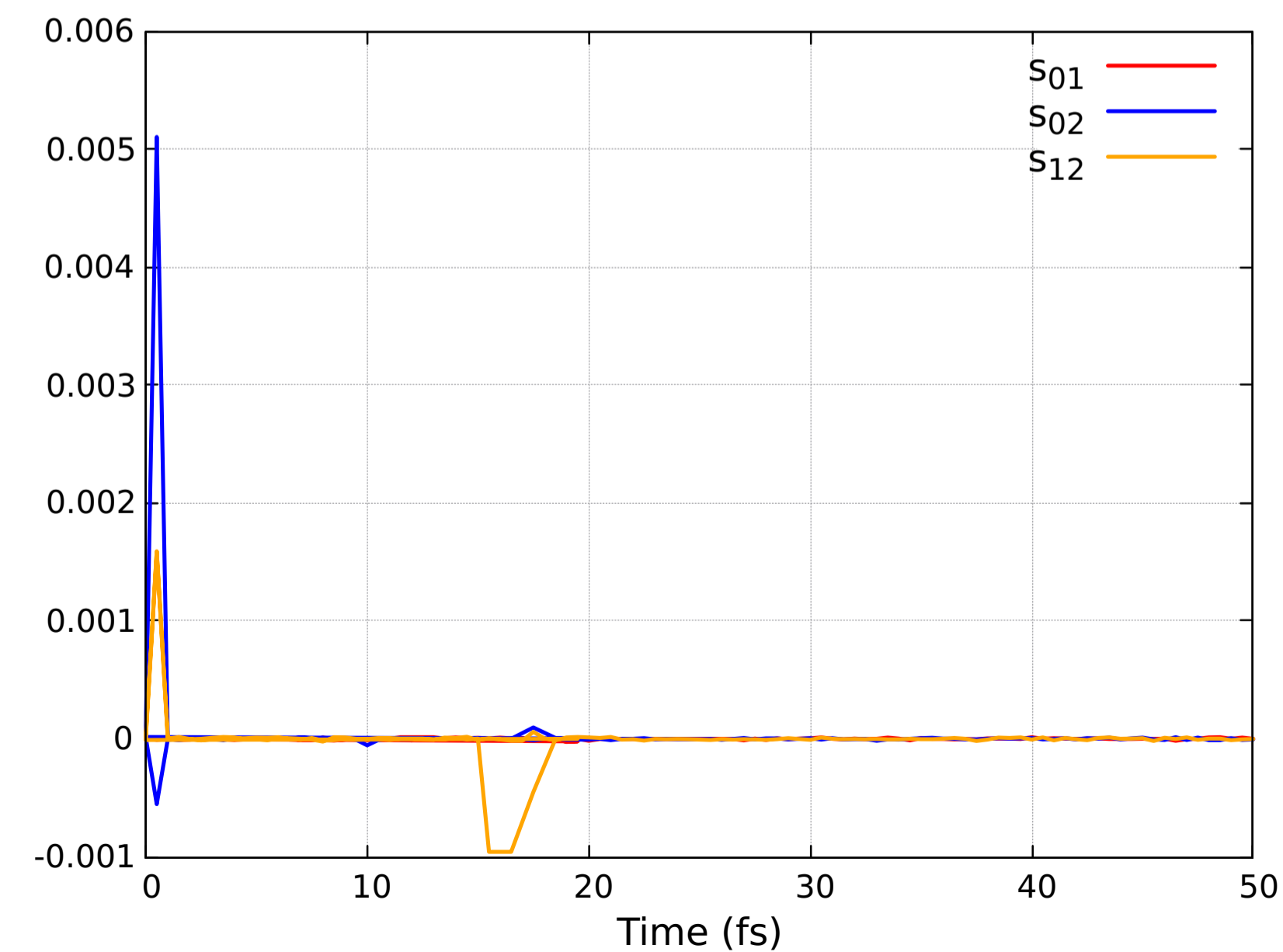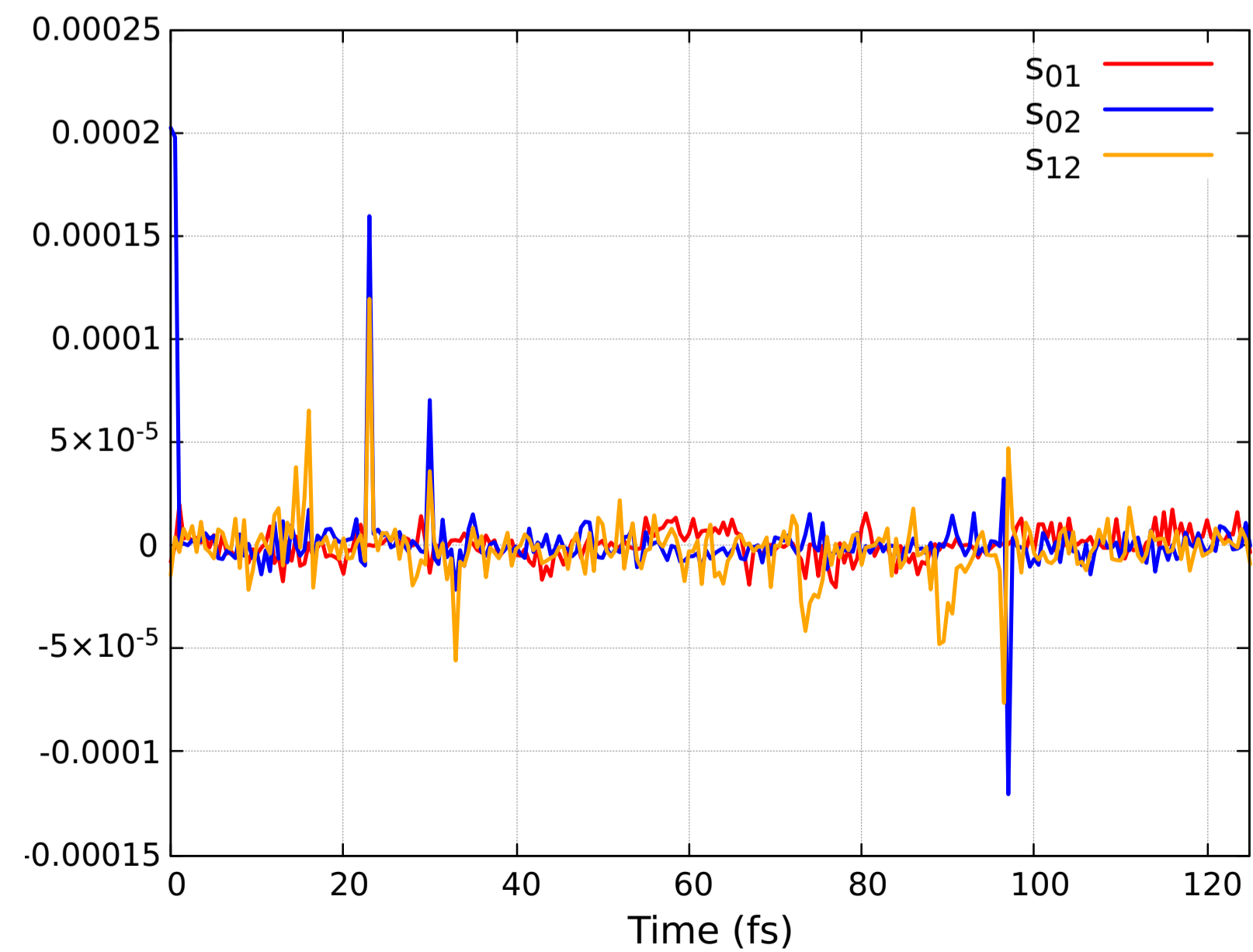

Supplement: SC-016-D4SC04987J-s008 [file SC-016-D4SC04987J-s008.pdf]

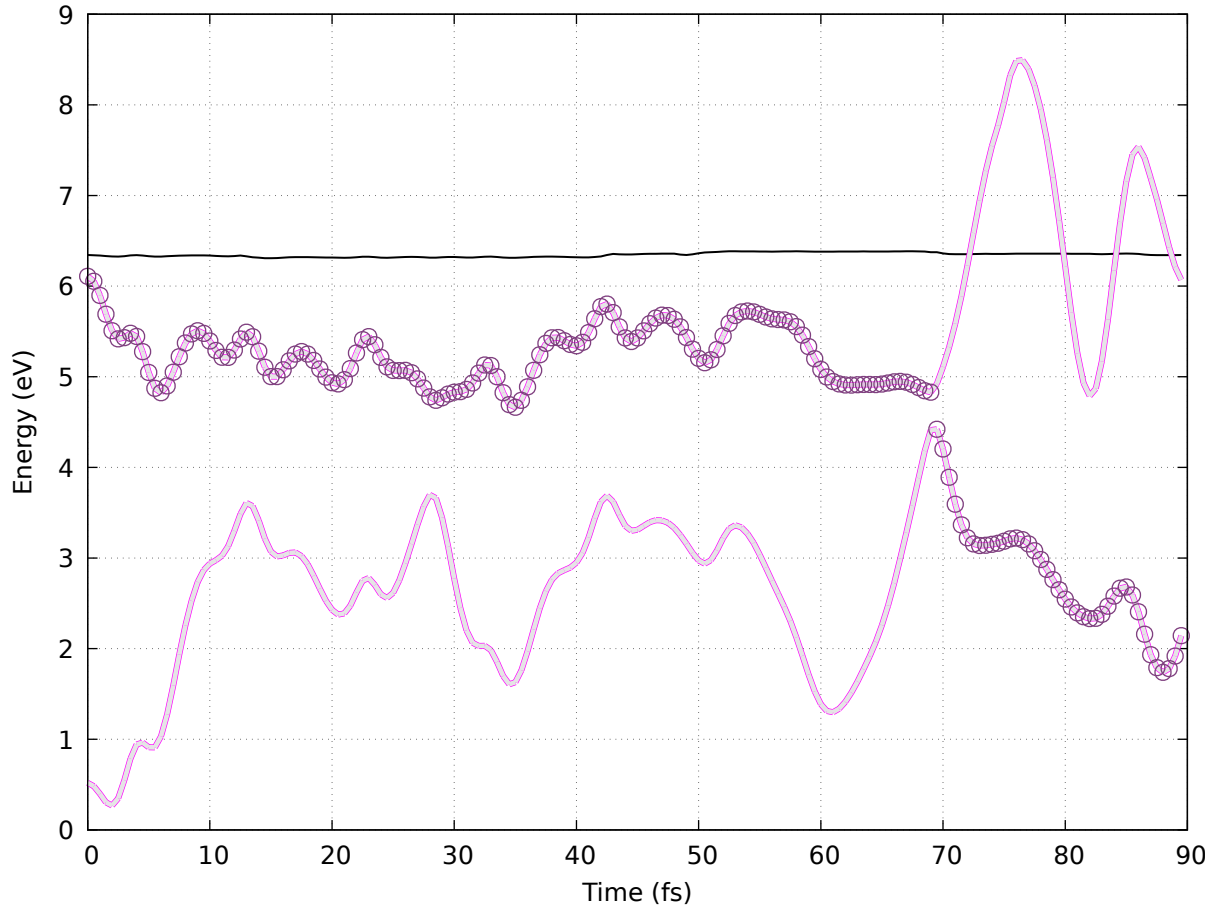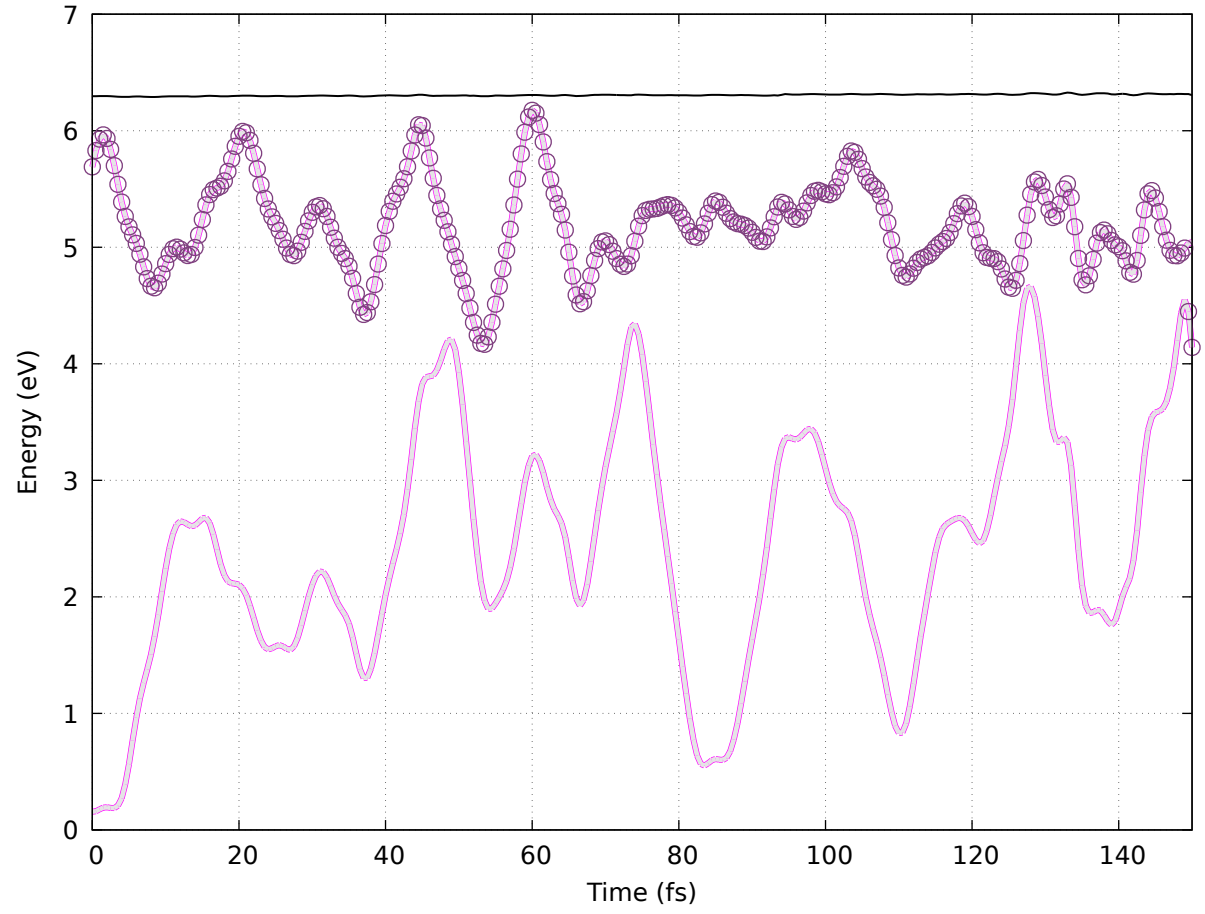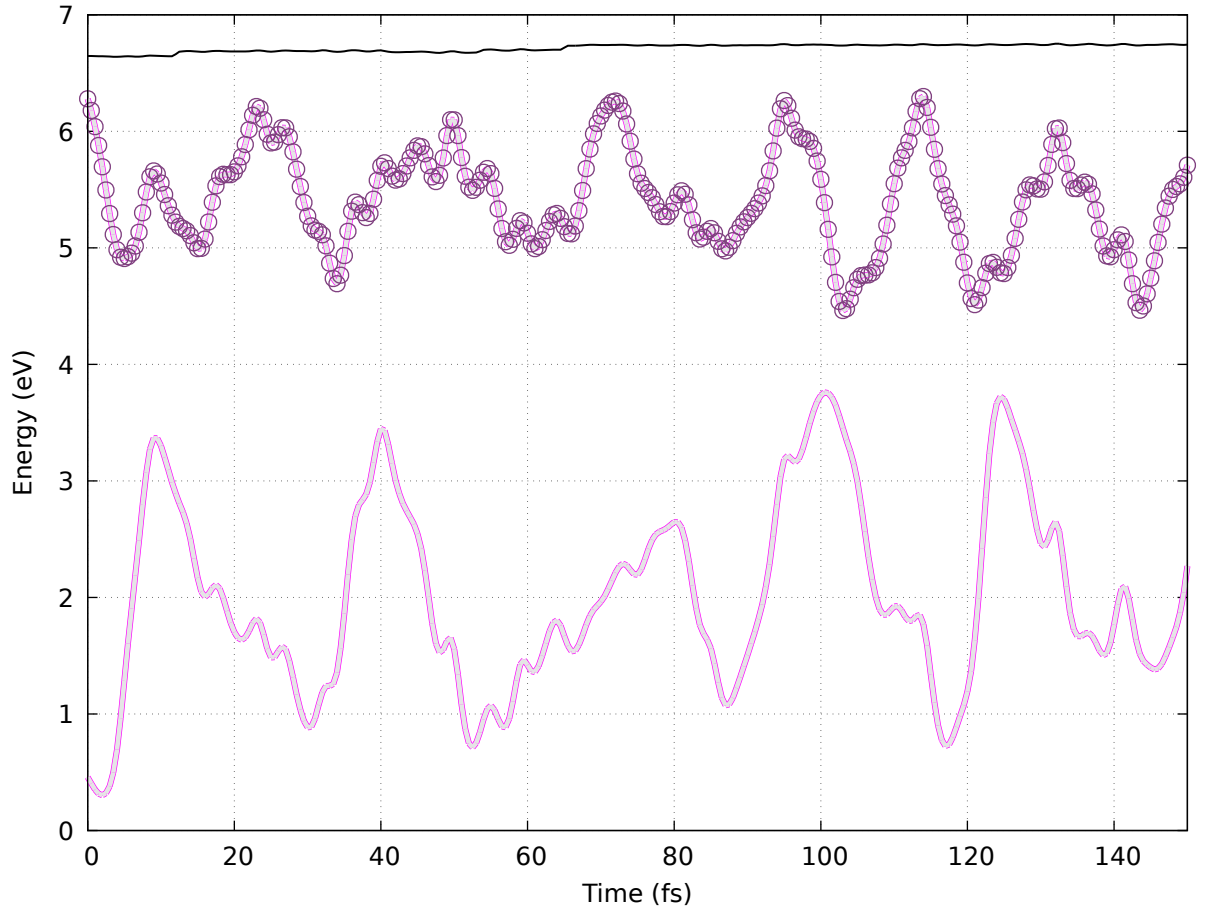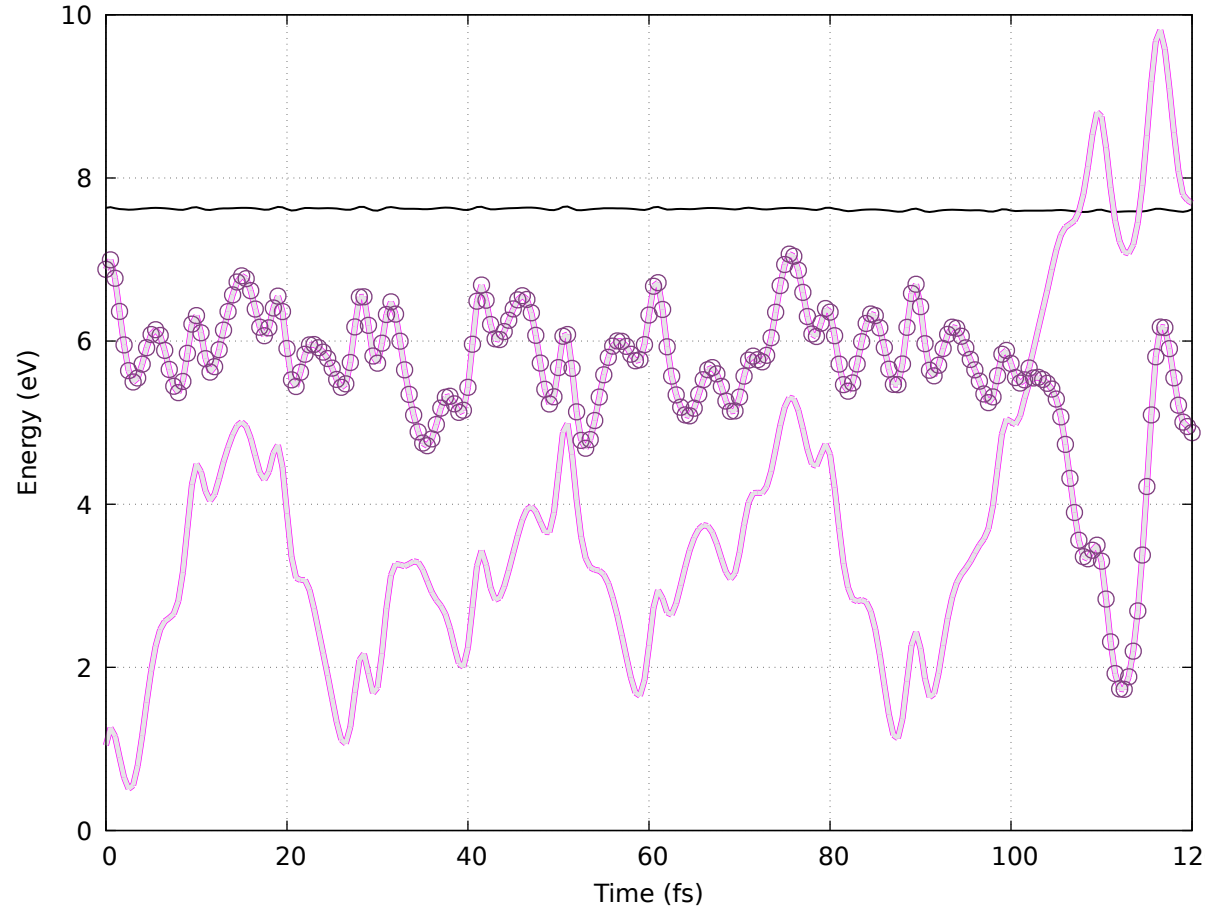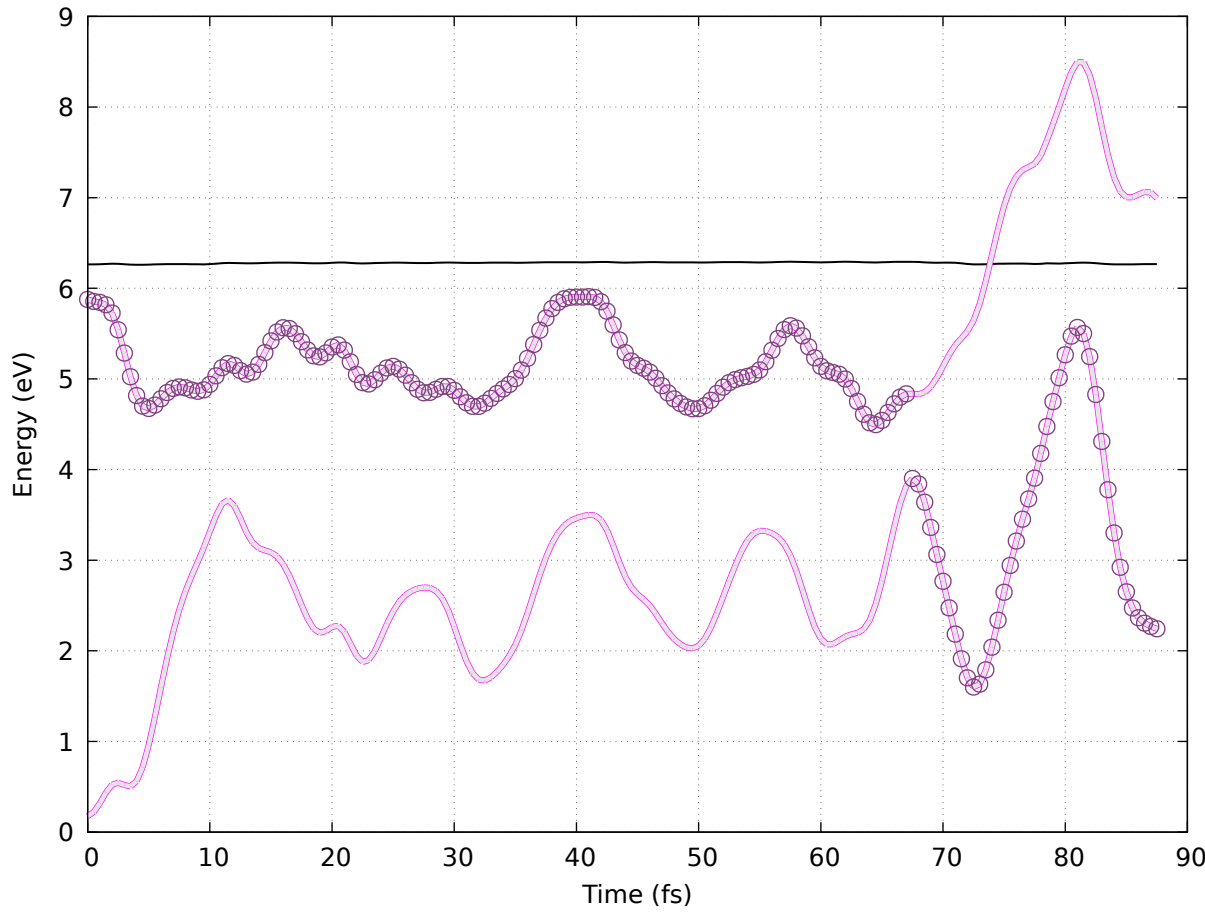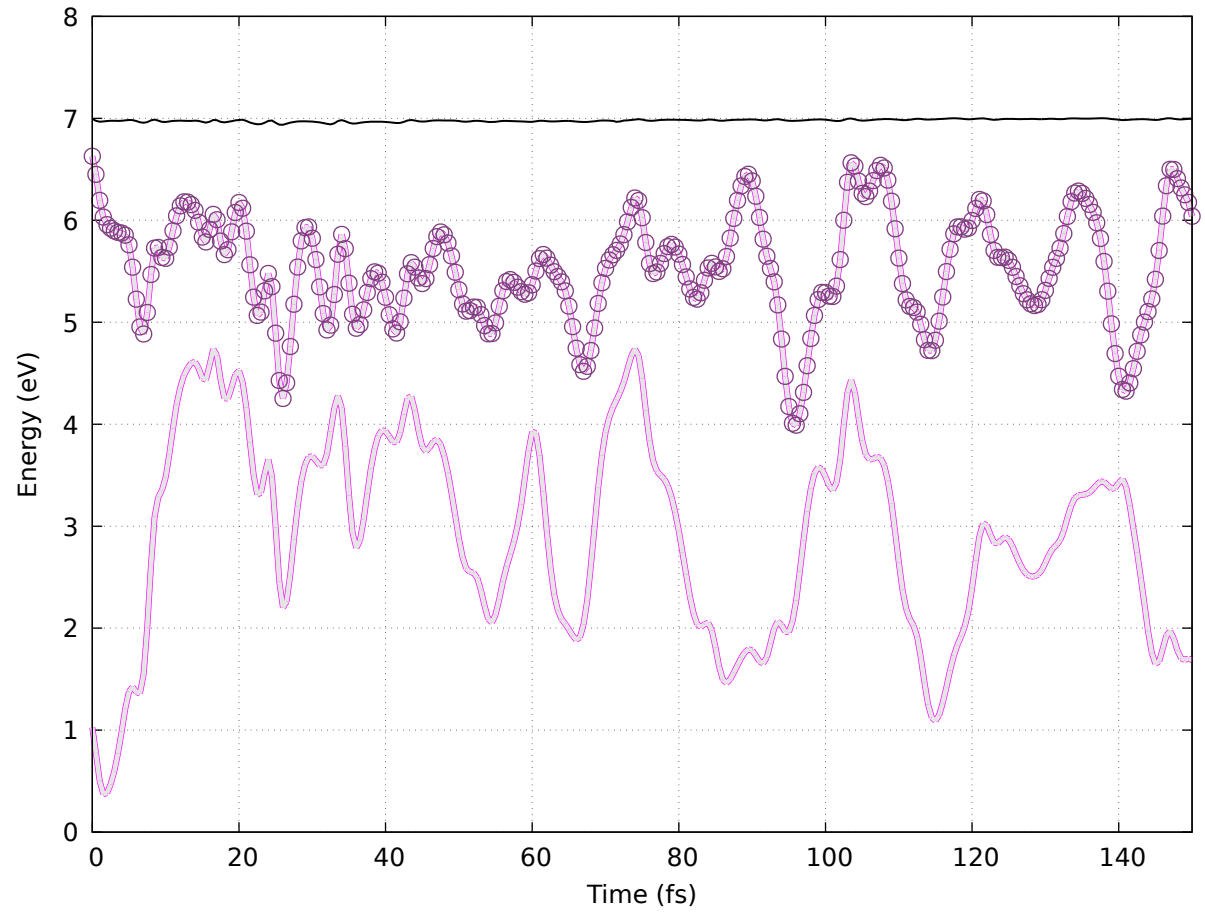

Supplement: SC-016-D4SC04987J-s009 [file SC-016-D4SC04987J-s009.pdf]
